# Deciphering distinct spatial alterations in N-glycan expression profiles in the spinal cord and brain of male rats in a neuropathic pain model

**DOI:** 10.1186/s11658-025-00709-7

**Published:** 2025-03-11

**Authors:** Hyun Jun Jang, Juhee Shin, Sangkyu Lee, Boyoung Lee, Dong Woon Kim

**Affiliations:** 1 Center for Cognition and Sociality, Institute for Basic Science, Daejeon, Republic of Korea; 2 Department of Oral Anatomy & Developmental Biology, Kyung Hee University College of Dentistry, Seoul, Republic of Korea

**Keywords:** Neuropathic pain, Spinal cord, Brain, N-glycan, MALDI MSI

## Abstract

**Background:**

Neuropathic pain is a complex condition resulting from damage or disease in the somatosensory nervous system, causing significant physical and emotional distress. Despite its profound impact, the underlying causes and treatment methods of neuropathic pain remain poorly understood.

**Methods:**

To better understand this condition, we conducted the first study examining the spatial distribution and dynamic expression changes of N-glycan molecules that play a crucial role in nervous system function and sustainable pain signal transmission across multiple regions of the spinal cord and brain in an experimentally induced neuropathic pain model, using matrix-assisted laser desorption/ionization mass spectrometry imaging (MALDI MSI).

**Results:**

Our findings revealed that neuropathic pain induces dynamic changes in N-glycan expression across various regions of the spinal cord and brain. Notably, we discovered distinct glycan profiles between the spinal cord and brain, with N-glycans downregulated in the spinal cord and upregulated in the brain at a time when mechanical allodynia is sustained following spinal nerve ligation (SNL). Significant changes in N-glycan expression were observed in the dorsal laminae IV/V/VI and the ventral horn of the spinal cord. Additionally, marked changes were detected in the contralateral regions of the primary sensory cortex (S1) and the primary sensory cortex hindlimb area (S1HL). Furthermore, we observed significant upregulation of N-glycan expression in the thalamus, anterior cingulate cortex (ACC), and medial prefrontal cortex (mPFC) in both ipsilateral and contralateral regions of the brain.

**Conclusions:**

Given that N-glycans are implicated in pain processing yet their precise role remains unclear, our study highlights the need to explore N-glycosylation with a more nuanced focus on both the spinal cord and brain. This research provides new insights into the mechanisms of persistent neuropathic pain and lays the groundwork for future studies and the development of targeted therapeutic strategies.

**Graphical Abstract:**

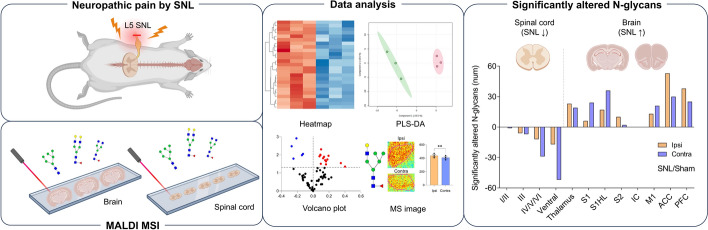

**Supplementary Information:**

The online version contains supplementary material available at 10.1186/s11658-025-00709-7.

## Background

Neuropathic pain is a chronic and debilitating condition resulting from damage or diseases affecting the somatosensory system, impacting 9.2% of the population, with most patients receiving inadequate and insufficient treatment [1]. Damage to peripheral nerves or the spinal cord is known to induce maladaptive plastic changes throughout the somatosensory nervous system, extending from the periphery to the cortex, often resulting in persistent pain [2, 3]. Previous studies suggest that neural circuit remodeling and structural synaptic plasticity within the “pain matrix” may play a more active role in chronic pain, rather than merely being a secondary effect of altered nociceptive signaling in the spinal cord [4–6]. Key regions within this matrix, such as the medial prefrontal cortex (mPFC), anterior cingulate cortex (ACC), primary (S1) and secondary (S2) somatosensory cortices, insular cortex (IC), and thalamus, are crucial not only in the perception of pain but also in its modulation, contributing to the emotional and cognitive aspects of neuropathic pain [7, 8]. These findings highlight neuropathic pain as a multilevel systems pathology. A deeper understanding of the molecular pathways driving neuropathic pain, from the spinal cord to various cortical regions, is essential for breaking down the complex pathophysiology of neuropathic pain and developing new biomarkers and more effective therapeutic targets. However, this crucial area of research remains largely underexplored.

N-glycosylation, the attachment of oligosaccharide chains to asparagine residues, is a vital posttranslational modification that significantly affects the structure, stability, and function of many proteins [9]. As a result, N-glycosylation profiles are increasingly recognized as both biomarkers and functional mediators in various diseases [10–13]. The brain undergoes frequent N-glycosylation to support complex neural communication, synaptic plasticity, development, and protection against damage. Recent studies have underscored the important role of N-glycans in the central nervous system, particularly owing to their impact on synaptic proteins involved in critical processes such as synaptic transmission and plasticity. Disruptions in N-glycosylation have therefore been associated with several neurodegenerative and neuropsychiatric disorders, including Alzheimer’s disease and schizophrenia [14, 15]. Additionally, recent research has underscored the potential role of glycosylation in the development and maintenance of chronic pain [16]. In animal models, abnormal glycosylation has been observed in spinal cord and DRG and shown to increase mechanical allodynia, thermal hyperalgesia, and reduce nociceptive behavior during the late phase of formalin-induced inflammatory pain [17]. Similarly, elevated levels of highly branched plasma N-glycans have been observed in patients with chronic low back pain [18]. Pharmacological inhibition of N-glycosylation has also been found to attenuate neuropathic pain behaviors, further suggesting that N-glycan modifications play a significant role in pain processing [19]. Although extensive evidence suggests the significant role of N-glycosylation in neuropathic pain, most studies have primarily focused on plasma or spinal cord levels, leaving the detailed spatial distribution within the brain largely unexplored. Therefore, gaining spatial insights into the distribution of N-glycans will be crucial for understanding their specific roles in the pathophysiology of neuropathic pain at both local and systemic levels throughout the nervous system.

To achieve these insights, in this study, we employed an effective approach for analyzing spatial N-glycan molecules using MALDI MSI. This mass spectrometry technique utilizes a laser and a matrix to ionize biomolecules on tissue surfaces, enabling the mapping of spatial distribution and relative quantification of molecules within tissues [20]. Recent studies using MALDI MSI analysis have revealed crucial roles of N-glycans in various pathological conditions, including cancer, neuroinflammation, and Alzheimer's disease [14, 21, 22]. Expanding upon these findings, we utilized MALDI MSI to investigate the spatial distribution of N-glycans in neural tissues specifically associated with neuropathic pain. We applied the spinal nerve ligation (SNL) model in male rats to induce neuropathic pain and investigated the changes in N-glycan expression within the spinal cord and brain regions involved in neuropathic pain processing. The SNL model effectively mimics clinical conditions such as radiculopathy and traumatic nerve injury, making it particularly valuable for exploring the mechanisms underlying the development and maintenance of neuropathic pain in both spinal and supraspinal regions. Our findings revealed distinct N-glycan signatures in the spinal cord and specific brain regions of neuropathic pain male rats compared with sham controls.

## Materials and methods

### Animals

All animal care and experimental studies were conducted in accordance with protocols approved by the Chungnam National University Institutional Animal Care and Use Committee (CNUH-2023-1A0014) and in accordance with the ethical guidelines of the National Institutes of Health and the International Association of Pain. Sprague–Dawley rats (7-week-old males, 180–200 g in weight) used in the experiment were purchased from Damul Science (Daejeon, Korea). Three rats per cage were housed in a controlled environment 12:12 h and free access to food and water in cages.

### Neuropathic pain rat model

Neuropathic pain was performed by spinal nerve ligation (SNL). The rats were acclimated for 1 week prior to the start of the experiment and the SNL model was established by following steps. Rats were anesthetized with 2% isoflurane in oxygen (Hana Palm, Seoul, Korea) and placed on a heating pad to maintain body temperature. For the injury, only the L5 nerve was separated and tied tightly twice with 3–0 silk thread to cause spinal nerve damage. In the sham group, the same surgical procedure was applied, except for the L5 nerve ligation.

### Immunohistochemistry

Animals (*n* = 5 per group) were deep-inhalation anesthetized and perfused with 2% heparinized phosphate-buffered saline (PBS, pH 7.4) and ice-cold 4% paraformaldehyde in PBS using a peristaltic pump at a rate of 20 mL/min. The lumbar region (L4–L6) of the spinal cord was immediately prepared and post-fixed in the same fixative overnight at 4 °C. Then, tissues were immersed in a series of sucrose solutions in PBS (from 10% to 30%) for cryoprotection. After 2 days, tissues were mounted on OCT compounds, sectioned at 30 µm-depth using a cryostat (CM1950; Leica, Wetzlar, Germany), and kept at 4 °C in storage buffer (30% glycerol, 30% ethylene glycol in PBS). For immunofluorescence staining, spinal sections were firstly incubated with a blocking buffer (5% normal serum/0.3% Triton X-100; Bio-Rad, Irvine, CA, USA) for 1 h to prevent nonspecific binding, and tissues were treated with the primary antibody each, Iba-1 (1:500, WAKO, 019–19741), GFAP (1:1000, WAKO, Z0334), and c-Fos (1:500, Abcam, ab190289), overnight at 4 °C. Next, to the tissues were added the single secondary antibodies conjugated with either Cy3 (1:400, Jackson ImmunoResearch, 111–165-003) or FITC (1:400, Jackson ImmunoResearch, 111–165-003) diluted in the same blocking buffer. The sections were counterstained with DAPI (1:5000, Waltham, B1098) for 5 min and then mounted on glass slides using gel mount solution (ProLong Gold antifade reagent). Images were obtained by using a ZEISS Axioscope 5 Smart Laboratory Microscope (CarlZeiss DE/Axio scope 5). To assess the immunostaining-positive area (cm^2^), the results of SNL were normalized by the results of sham.

### Quantitative real-time PCR

Ipsilateral spinal cord tissues from all groups (*n* = 4 per group, L4–L5 segment, 0.7 cm) were rapidly homogenized and isolated in TRIzol reagent (Invitrogen, Carlsbad, CA, USA). Complete DNA (cDNA) synthesis was then carried out in a 20 µl reaction using TOPscript RT DryMix (Enzynomics, Daejeon, Republic of Korea). Quantitative Real-time PCR (QPCR) was performed with the following conditions: 95 °C for 10 min, then 40 amplification cycles of 95 °C for 15 s and 60 °C for 1 min, using an AriaMx Realtime PCR system (Agilent Technologies, Santa Clara, CA). Supplementary Table 1 presents the primer sequences (Cosmogenetech, Seoul, Republic of Korea) used in the PCR. The mRNA levels of each target gene were normalized to that of GAPDH mRNA. The fold change of the mRNA level was calculated by using the 2^−ΔΔCt^ method as described previously [23].

### MALDI MSI sample preparation

For sample preparation for MALDI MSI of N-glycans, refer to the previous study [24]. Fresh-frozen spinal cord and brain (*n* = 3 per group) were cut into coronal sections at thickness of 12 μm using a CM1950 cryostat (Leica, Wetzlar, Germany) and then thaw-mounted on conductive indium tin oxide (ITO) slides (Applied Surface Tech Asia, Gyeonggi-do, Republic of Korea). Both spinal cord and brain sections were then incubated at 60 °C for 1 h. Subsequently, sections were washed with ethanol and distilled water (DW). Citraconic acid buffer (pH 3.0) was used for 20 min of antigen retrieval (AR), followed by 10 min of room-temperature cooling. The tissue slide was put in distilled water (DW) after the procedure of substituting half of the AR buffer with DW three times. PNGase F enzyme (PRIME-LY™ ULTRA GLYCOSIDASE, N-zyme Scientifics, PA, USA) was applied to all slides under the same conditions using an M3+ sprayer (HTX technologies, NC, USA) at a concentration of 0.1 mg/ml, with a nozzle temperature of 45 °C, pressure of 10 psi, flow rate of 25 µL/min, nozzle velocity of 1200 mm/min, track spacing of 3 mm, and number of passes set to 15. The sample slides were placed in a chamber maintained at 97% relative humidity using 150 g/L K_2_SO_4_ and incubated at 37 °C for 2 h. α-Cyano-4-hydroxycinnamic acid (7 mg/ml, CHCA; Sigma Aldrich, USA) in 50% acetonitrile was applied using an M3+ sprayer with a nozzle temperature of 80 °C, pressure of 10 psi, flow rate of 100 µL/min, nozzle velocity of 1300 mm/min, track spacing of 2.5 mm, and number of passes set to 10. The performance of the M3+ sprayer was confirmed by the uniform application of the matrix on the slides (Supplementary Fig. 1).

### MALDI MSI measurement

N-glycan images of the mouse spinal cord and brain were obtained using a MALDI QTOF instrument, timsTOF fleX (Bruker Daltonik GmbH, Bremen, Germany) equipped with a 10-kHz SmartBeam 3D laser (355 nm wavelength). Mass spectra were recorded using 500 laser shots per each pixel in positive ion mode. The mass range was set to 900–3500 Da. The laser focusing size and spatial resolution were set to 30 μm for spinal cord tissue sections and 100 μm for brain tissue sections. Mass calibration was performed using ESI tuning mix (Agilent, CA, USA) and peptide calibration standard (Bruker Daltonik GmbH, Bremen, Germany).

### Data analysis

The regions of interest (ROIs) in the male rat's spinal cord and brain were defined based on two anatomical atlas references [25, 26]. All statistical analyses were performed using GraphPad Prism (version 10.2.0). Statistical significance between two groups was assessed using unpaired or paired *t*-tests, while comparisons among multiple groups were conducted using two-way analysis of variance (ANOVA) followed by Tukey’s post hoc test. *p*-Values < 0.05 were considered statistically significant. Data are expressed as mean ± standard error of the mean (SEM). Measured mass spectra were normalized to total ion count (TIC) and converted to mass spectrometry images using SCiLS Lab 2023a software (Bruker Daltonik GmbH, Bremen, Germany). All raw data for N-glycan peaks obtained from MALDI MSI measurements are provided in Additional File 2 as Supplementary Table 6. Extracted data from SCiLS software were analyzed using GraphPad Prism (version 10.2.0) for *t*-tests and volcano plots, and uploaded to MetaboAnalyst (version 6.0) for partial least squares-discriminant analysis (PLS-DA), variable importance in projection (VIP) analysis, and hierarchical clustering heatmaps. Data were filtered using a variance filter (standard deviation) and abundance filter (mean intensity value), and autoscaled (mean-centered and divided by the standard deviation of each variable). The heatmap was clustered using the Ward method, with distances measured by Euclidean analysis. The volcano plot was constructed utilizing log_2_(fold change) values and -log_10_(*p*-values) The *p*-values were derived from two-tailed unpaired *t*-tests for sham and SNL comparisons, and from two-tailed paired *t*-tests for ipsilateral and contralateral comparisons. The structure of the proposed N-glycan was annotated using GlycoWorkBench on the basis of previously published studies [27, 28]. Supplementary Table 2 contains a list of all N-glycans reported in this study along with proposed N-glycan structures. The relative abundance percentage for each type of N-glycan was calculated as the sum of the relative intensities of N-glycans within that type, divided by the total sum of the relative intensities of all N-glycans.

## Results

### Neuropathic pain injury induced glial activation and neuroinflammation in the ipsilateral spinal cord

**Fig. 1:**
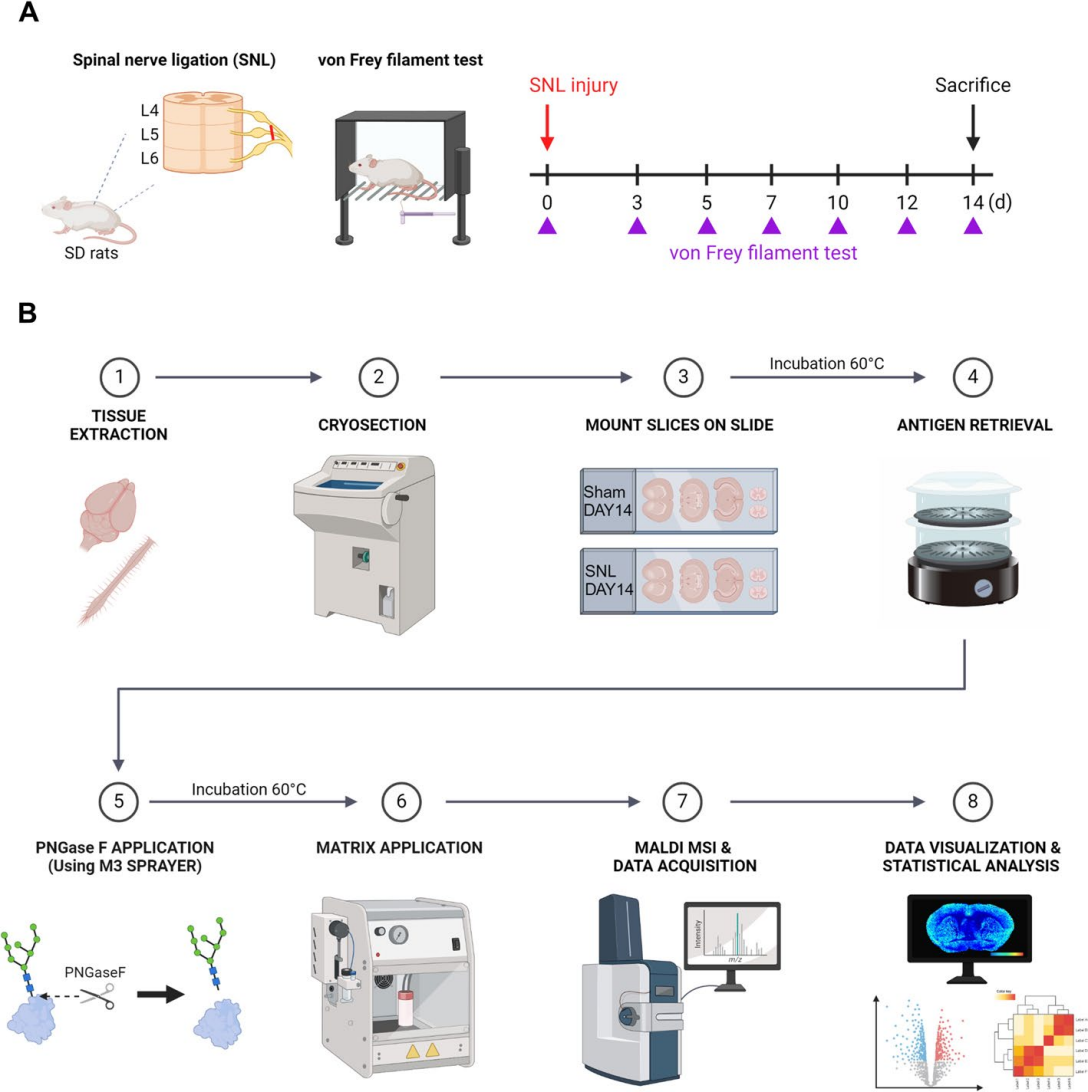
Schematic description of the neuropathic pain model and tissue preparation for MALDI MSI.** A** Neuropathic pain was induced in rats with lumbar 5 (L5) spinal nerve ligation (SNL). Prior to SNL surgery, rats were subjected to a von Frey filament test for baseline (> 10 g) on day 0. Following surgery, the von Frey test was repeated on day 3, 5, 7, 10, 12, and 14. On day 14, the brain and spinal cord were harvested for immunohistochemistry, mRNA analysis, and MALDI MSI. **B** Sample preparation for N-glycan measurement and analysis via MALDI MSI. Detailed methods for sample preparation are described in the “Materials and methods” section

**Fig. 2:**
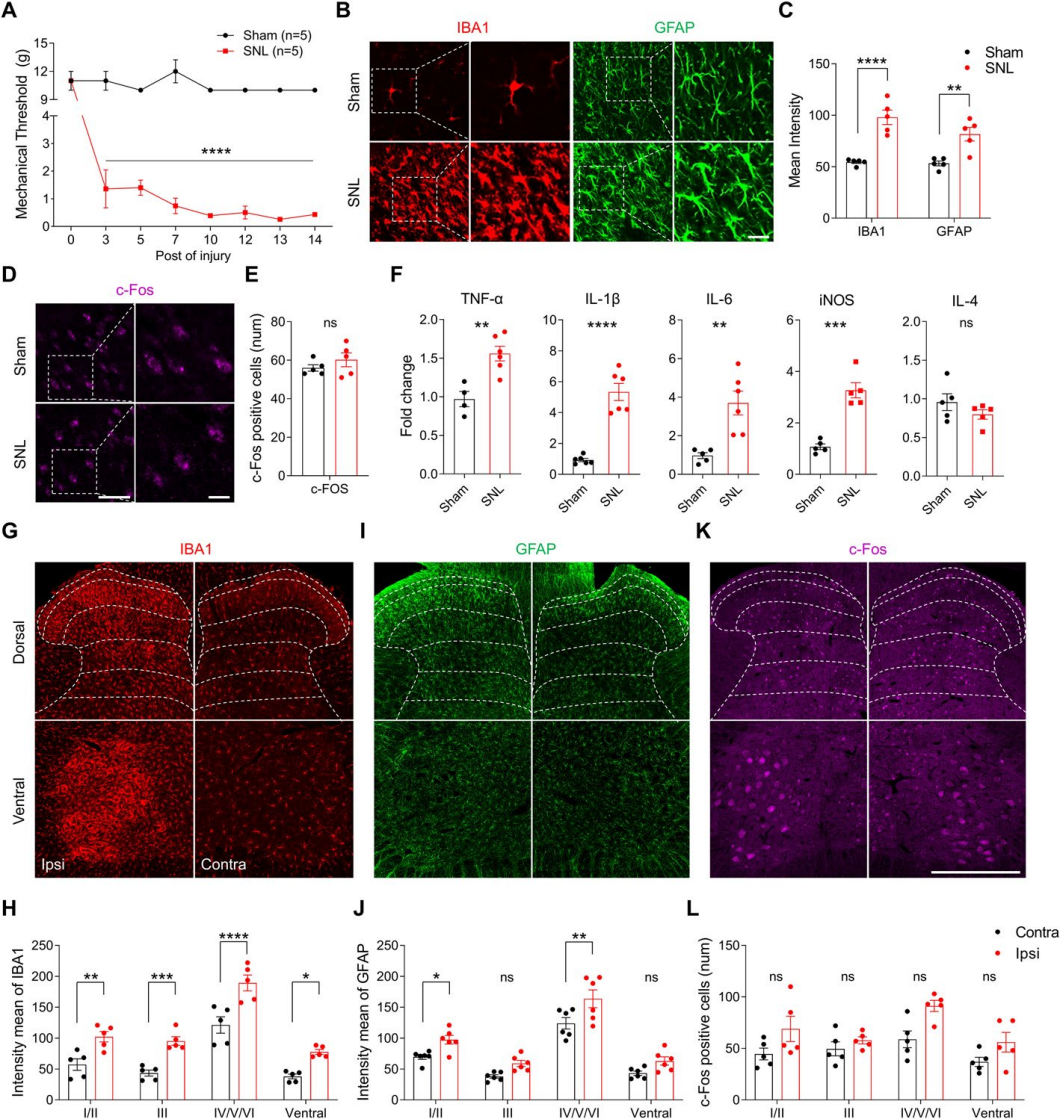
Neuropathic pain injury induces glial activation and neuroinflammation in the ipsilateral spinal cord.** A** Pain sensitivity was assessed using the von Frey filament test to measure mechanical thresholds from baseline (day 0) to day 14 post-injury (two-way ANOVA, ****p < 0. 001, *n* = 5 mice per group) **B-E** Representative confocal images of the ipsilateral superficial dorsal horn (laminae I/II) in the sham and SNL groups. Tissues were stained for IBA1 (microglia, **B**), GFAP (astrocytes, **B**), and c-Fos (neurons, **D**), with data presented at both low (left, scale bar = 50 μm) and high magnification (right, scale bar = 20 μm). Quantification of the mean intensity of IBA1 and GFAP (**C**) and the number of c-Fos-positive cells (**E**) in the sham and SNL groups (unpaired *t*-test, ***p < 0.001, ****p < 0.0001, error bars: SEM,* n* = 5 mice per group). **F** The ipsilateral dorsal horn of sham and SNL rats was analyzed for mRNA expression of pro-inflammatory cytokines (TNF-α, IL-1β, IL-6, iNOS, and IL-4) via qRT-PCR (unpaired *t*-test, *p < 0.05, **p < 0.01, ***p < 0.001, error bars: SEM,* n* = 5–6 mice per group). **G**, **I**, **K** Representative confocal images of the contralateral (Contra) and ipsilateral (Ipsi) dorsal and ventral horns of the spinal cord in SNL rats. Tissues were stained for IBA1 (**G**), GFAP (**I**), and c-Fos (**K**), with dashed lines indicating the laminae (I-VI) layers of the spinal cord. **H**, **J** Quantification of the mean intensity of IBA1 (**H**) and GFAP (**J**) (unpaired *t*-test, *p < 0.05, **p < 0.01, ***p < 0.001, error bars: SEM, scale bar = 250 μm,* n* = 5–6 mice per group). **L** Comparison of the number of c-Fos-positive cells between superficial (I/II) and deep layers (III/IV/V/VI) in each ipsilateral and contralateral side (unpaired *t*-test, n.s. = nonsignificant, error bars: SEM). Data are representative of *n* = 5–6 independent experiments

To focus on understanding the central processing of pain signals by examining the spatial distribution and dynamic expression of N-glycans in the spinal cord and brain, we established a neuropathic pain animal model using L5 spinal nerve ligation (SNL) (Fig. 1A). The SNL model in rodents is a widely used and well-characterized method for studying neuropathic pain resulting from peripheral nerve injury [29]. Three days post-surgery, the mechanical threshold significantly decreased and remained at a low level until day 14 post-injury (Fig. 2A). To compare and analyze N-glycan expression in the spinal cord and brain that contribute to persistent mechanical allodynia in neuropathic pain, we selected a time point of approximately 2 weeks, when the pain is sustained, rather than an earlier time frame of 3–7 days when pain is primarily inducible. In addition to behavioral changes that establish the pain phenotype, we observed physiological and molecular evidence, including glial activation, which is widely recognized as an important aspect of central sensitization and pain persistence [30].

On day 14, glial activation was confirmed by immunohistochemistry using IBA1 and GFAP, specific markers for microglia and astrocytes, respectively. Following the nerve injury, reactive microglia and astrocytes were found in the ipsilateral dorsal horn of spinal cord in the SNL group compared with the sham group as evidenced by morphological changes and increased Iba1 and GFAP intensity in the superficial dorsal horn (laminae I/II) (Fig. 2B, C). Additionally, neuronal activity was assessed using c-Fos, a marker that reflects spinal nociceptive processes, and we found no significant differences of the numbers of c-Fos-labeled neurons between the groups (Fig. 2D, E). Given the glial activation, we observed an upregulation of pro-inflammatory genes, including TNF-α, IL-1β, IL-6, and iNOS, which are the key cytokines secreted by activated glial cells that contribute to secondary injury. In contrast, no changes were observed in the expression of the anti-inflammatory genes IL-4 (Fig. 2F).

The spinal cord is organized into distinct laminae layers, each playing a crucial role in processing sensory and motor information: Lamina I is composed mainly of neurons that respond to high-threshold C fibers and Aδ fibers [31, 32]; Lamina II, the substantia gelatinosa, plays a key role in modulating nociceptive (pain-related) signals. After injury, lamina II undergoes significant plastic changes, which can lead to altered pain perception [33]; In lamina III, after injury, there is an abnormal overprojection of Aβ fibers into lamina II, resulting in pain hypersensitivity and altered sensory perception [34, 35]; Laminae IV/V/VI, also known as the nucleus proprius, are involved in processing tactile and proprioceptive information, as well as the transmission of both pain and nonpain signals to higher centers such as the brainstem and thalamus [36]. The ventral horn is primarily responsible for motor functions, and it plays transmitting motor signals from the central nervous system to skeletal muscles via the somatic motor neurons [37]. Therefore, we compared glial and neuronal activation through the spinal layers to understand the transmission of pain signals from the spinal cord to the brain in ipsilateral or contralateral SNL rats.

First, microglia were activated in all the layers of the spinal cord on the ipsilateral side, suggesting that microglia play a central role in responding to pain injury (Fig. 2G, H). In contrast, activated astrocytes were observed primarily in laminae I/II and lamina IV/V/VI of the ipsilateral side compared with the contralateral side (Fig. 2I, J). Our data also showed that there was no difference compared with the contralateral side (Fig. 2K, L). Taken together, these findings suggest that microglia and astrocytes contribute to the development of neuropathic pain, with differential involvement across spinal cord laminae layers and highlight the complex interaction between glial activation and neuronal activity in driving persistent pain signaling.

### Neuropathic pain injury induced only weak changes in N-glycan profiles in laminae I/II and III of the spinal dorsal horn

**Fig. 3:**
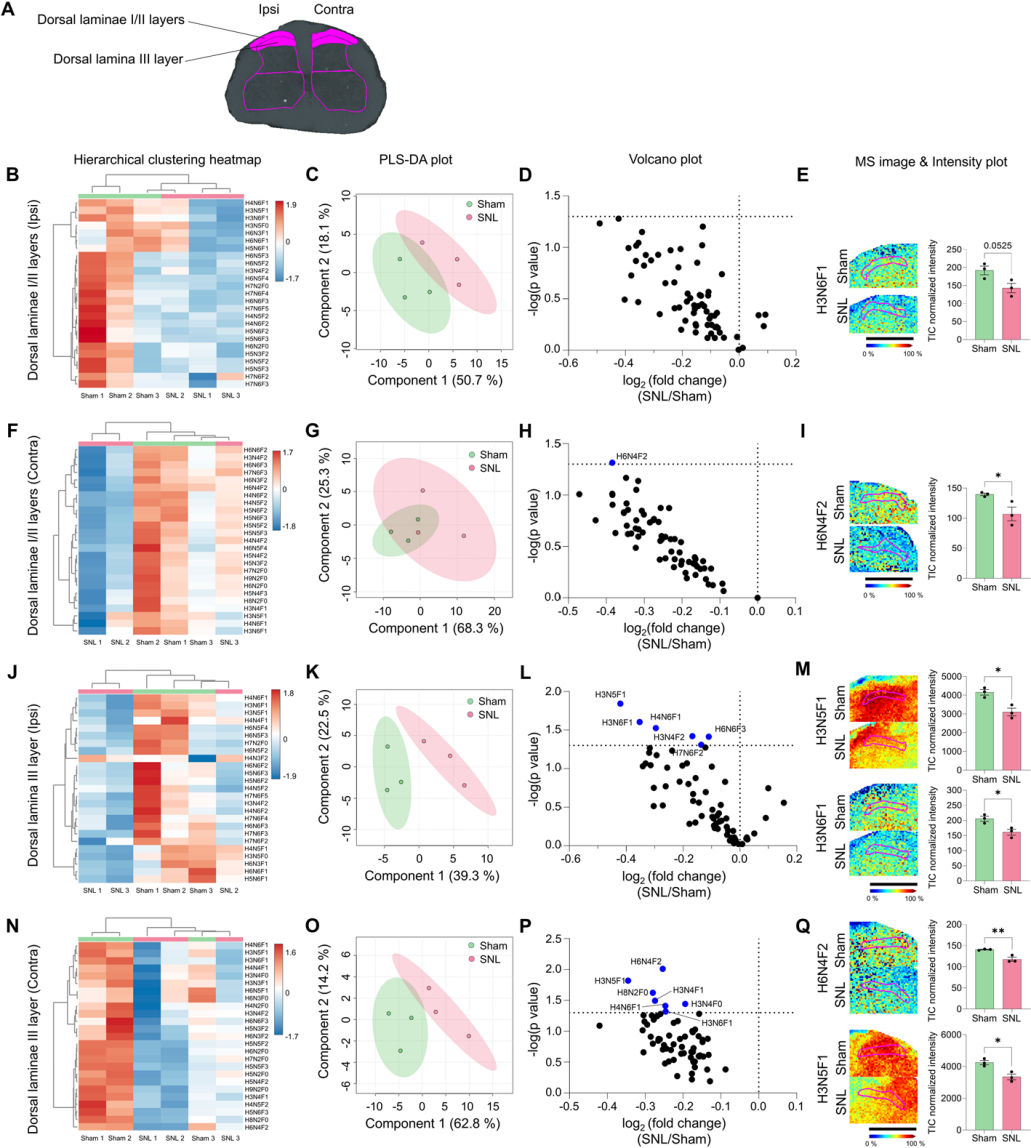
N-glycan expression shows minimal changes in dorsal laminae I/II and dorsal lamina III layers of the spinal cord following SNL, with reduced expression. **A** ROI of dorsal laminae I/II and dorsal lamina III layers. **B–Q** Hierarchical clustering heatmap, PLS-DA plot, volcano plot, and MALDI mass spectrometry images with intensity plot of ipsilateral and contralateral dorsal laminae I/II layers (**B**–**I**) and dorsal lamina III layer (**J**-**Q**). The correlation of the top 25 N-glycans detected in three samples from sham (green) and SNL (red) groups is shown in the hierarchical clustering heatmap (H: hexose, N: N-acetylglucosamine, and F: fucose). The PLS-DA plot of N-glycans from sham and SNL groups with a 95% confidence region. In the volcano plot, blue dots indicate N-glycans significantly decreased in the SNL group compared with the sham group (*p* < 0.05). Representative MALDI mass spectrometry images and intensity plots of N-glycans demonstrate significant differences between sham and SNL groups (unpaired *t*-test, *p < 0.05, **p < 0.01, error bars: SEM). Data are representative of *n* = 3 independent experiments. Scale bars = 1 mm

To investigate alterations in N-glycan expression pattern in the dorsal horns of the spinal cord following SNL, we performed MALDI MSI for N-glycans and divided the dorsal horn into three ROIs: laminae I/II, III, and IV/V/VI. Since laminae I/II serve as the primary termination zones for nociceptive primary afferents, and lamina III relays Aβ afferent input to lamina I nociceptive projection neurons, both of which have been implicated in neuropathic pain [38], we initially examined N-glycan expression in these areas. We obtained three serial 12-µm spinal cord slices at L5 from each animal, which had been confirmed to exhibit behavioral allodynia 14 days post-SNL, and processed them for MALDI MSI (Fig. 1B). Through MALDI MSI analysis of the spinal cord, we detected 65 N-glycans in the rat spinal cord (Supplementary Table 2). First, we compared the expression patterns between sham and SNL groups in both ipsilateral and contralateral laminae I/II (Fig. 3A). The hierarchical clustering heatmap (hereafter referred to just as heatmap) and PLS-DA plot did not show clear clustering in the ipsilateral laminae I/II (Fig. 3B, C). We proceeded with a volcano plot, which did not reveal any significant changes in N-glycan expression (Fig. 3D, E). Similarly, in the contralateral dorsal laminae I/II, the heatmap and PLS-DA plot showed no distinct clustering between the sham and SNL groups (Fig. 3F, G). The volcano plot identified one N-glycan that exhibited a significant decrease in the SNL group compared with the sham group (Fig. 3H, I).

Similarly, in the ipsilateral and contralateral dorsal lamina III, heatmaps and PLS-DA plots showed no distinct clustering between the sham and SNL groups (Fig. 3J, K, N, O). The volcano plots identified six and seven N-glycans with significant decreases in the SNL group compared with the sham group on the ipsilateral and contralateral sides, respectively (Fig. 3L, M, P, Q). Representative images of significant N-glycans were selected based on the VIP scores that contributed to PLS-DA clustering (Fig. 3E, I, M, Q, and Supplementary Fig. 2A, B, 3A–D). Overall, these data suggest that laminae I/II and III showed no distinct changes in N-glycan expression on either the ipsilateral or contralateral side 14 days after SNL. In addition, there were no significant differences in N-glycan expression between the ipsilateral and contralateral sides within either the sham or SNL groups in laminae I/II and III even though the SNL was applied only to the left L5, suggesting no lateralized effects (Supplementary Fig. 2C, D, 3E, F). Although there were few significant alterations in N-glycans following SNL, the relative intensities of most N-glycans decreased in the SNL group compared with the sham group (Fig. 3D, E, H, I, Supplementary Fig. 2A, B, and Supplementary Table 3).

### Neuropathic pain injury induced significant reduction in N-glycan expression in laminae IV/V/VI in the spinal dorsal horn

**Fig. 4:**
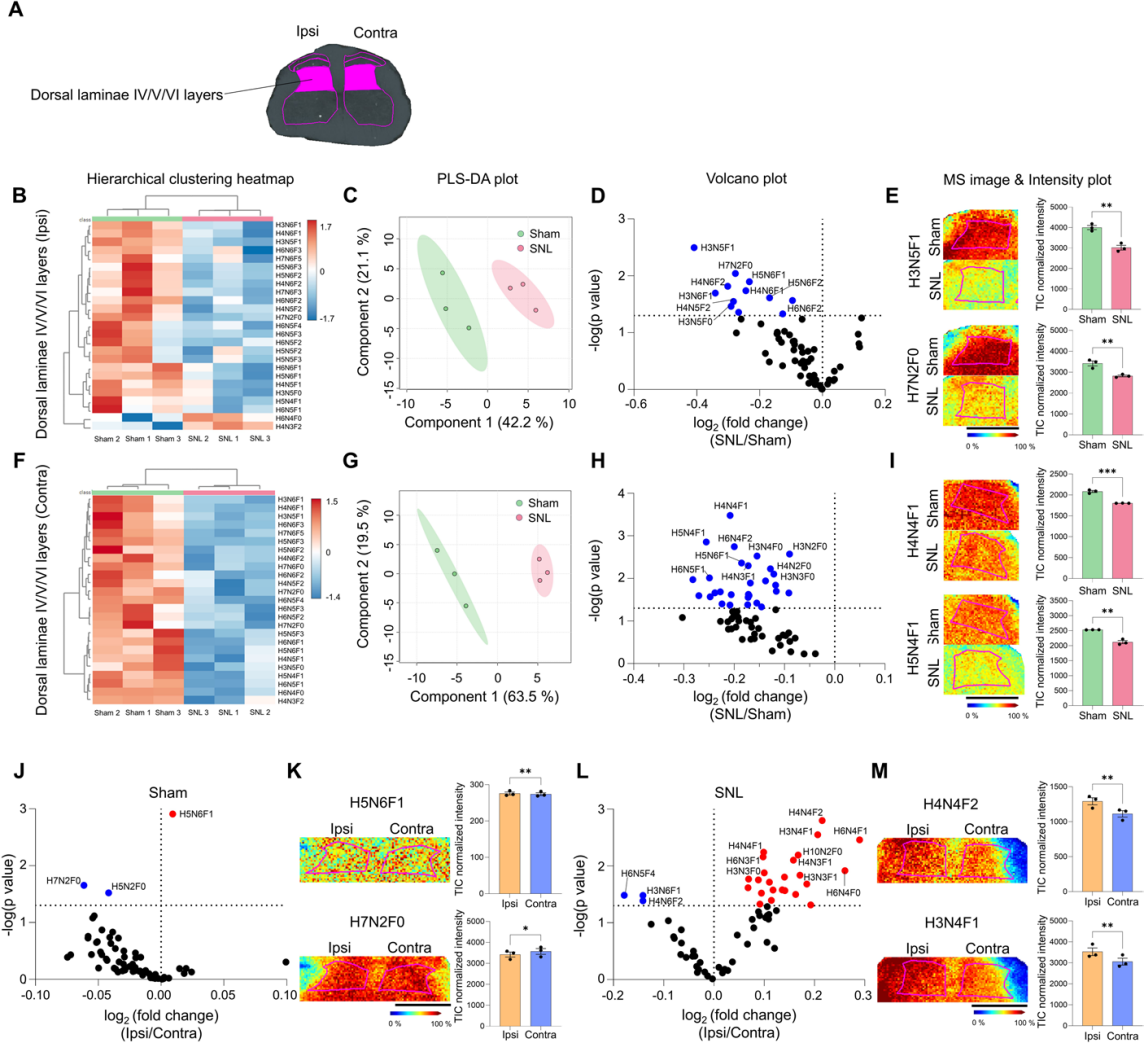
N-glycan expression significantly decreases in the dorsal laminae IV/V/VI layers following SNL. **A** ROI in the dorsal laminae IV/V/VI layers. **B**–**I** Hierarchical clustering heatmap, PLS-DA plot, volcano plot, and MALDI mass spectrometry images with intensity plots of ipsilateral (**B**–**E**) and contralateral (**F**–**I**) dorsal laminae IV/V/VI layers, comparing sham and SNL groups. The correlation of the top 25 N-glycans detected in three samples from sham (green) and SNL (red) groups is shown and presented in the hierarchical clustering heatmap (H: hexose, N: N-acetylglucosamine, and F: fucose). The PLS-DA plot of N-glycans of sham and SNL groups with 95% confidence region. In the volcano plot, the blue dots indicate N-glycans significantly decreased in the SNL group compared with the sham group (*p* < 0.05). Representative MALDI mass spectrometry images and intensity plots of N-glycans reveal significant differences between sham and SNL groups (unpaired *t*-test, **p < 0.01, ***p < 0.001, error bar: SEM). **J**–**M** Volcano plots and MALDI mass spectrometry images with intensity plot of ipsilateral (Ipsi, orange) and contralateral (Contra, blue) dorsal laminae IV/V/VI layers of sham (**J**, **K**) and SNL (**L**, **M**) group. In the volcano plot, blue dots represent N-glycans significantly decreased on the ipsilateral side compared with the contralateral side, while red dots represent N-glycans significantly increased on the ipsilateral side compared with the contralateral side (*p* < 0.05). Representative MALDI mass spectrometry images and intensity plots of N-glycans highlight significant differences between the ipsi and contra sides within each group (paired *t*-test, *p < 0.05, **p < 0.01, error bar: SEM). Data are representative of *n* = 3 independent experiments. Scale bars = 1 mm

Although the dorsal laminae IV/V/VI layers primarily receive direct Aβ fiber input from primary afferents, wide dynamic range (WDR) projection neurons concentrated in these laminae also receive polysynaptic inputs that relay nociceptive information from the superficial laminae to the central nervous system [39]. Notably, the laminae IV/V/VI layers (Fig. 4A) exhibited clear differences between the sham and SNL groups on both the ipsilateral and contralateral sides, as shown by heatmap and PLS-DA analyses (Fig. 4B, C, F, G). We identified the most significant reductions in N-glycans within the spinal dorsal horn, with 12 reductions on the ipsilateral side and 29 on the contralateral side out of 65 N-glycans in the volcano plot (Fig. 4D, E, H, I and Supplementary Fig. 4A-D).

Additionally, differential analysis of N-glycan expression between the ipsilateral and contralateral dorsal laminae IV/V/VI layers within the SNL group revealed hemisphere-specific variations, which were less pronounced in the sham group (Fig. 4J–M and Supplementary Fig. 4E, F). Twenty-seven N-glycans showed significant changes in the comparison between ipsilateral and contralateral sides of SNL, with three N-glycans decreased on the ipsilateral side and 24 N-glycans increased (Fig. 4L and Supplementary Table 5). To further investigate lateralization effects within the SNL group, we aimed to determine whether the significant differences observed on the ipsilateral side were due to dynamic changes in N-glycan expression specifically on the ipsilateral side or if they may also be influenced by alterations on the contralateral side. By comparing these changes with the sham group, we found that most of the significant N-glycan alterations were downregulated on the contralateral side, with minimal changes observed on the ipsilateral side (Supplementary Fig. 5A, B). This suggests that the lateralization effects may be driven primarily by dynamic changes in the contralateral dorsal laminae IV/V/VI following SNL.

### Neuropathic pain injury induced significant reductions in N-glycan expression in the spinal ventral horn

**Fig. 5:**
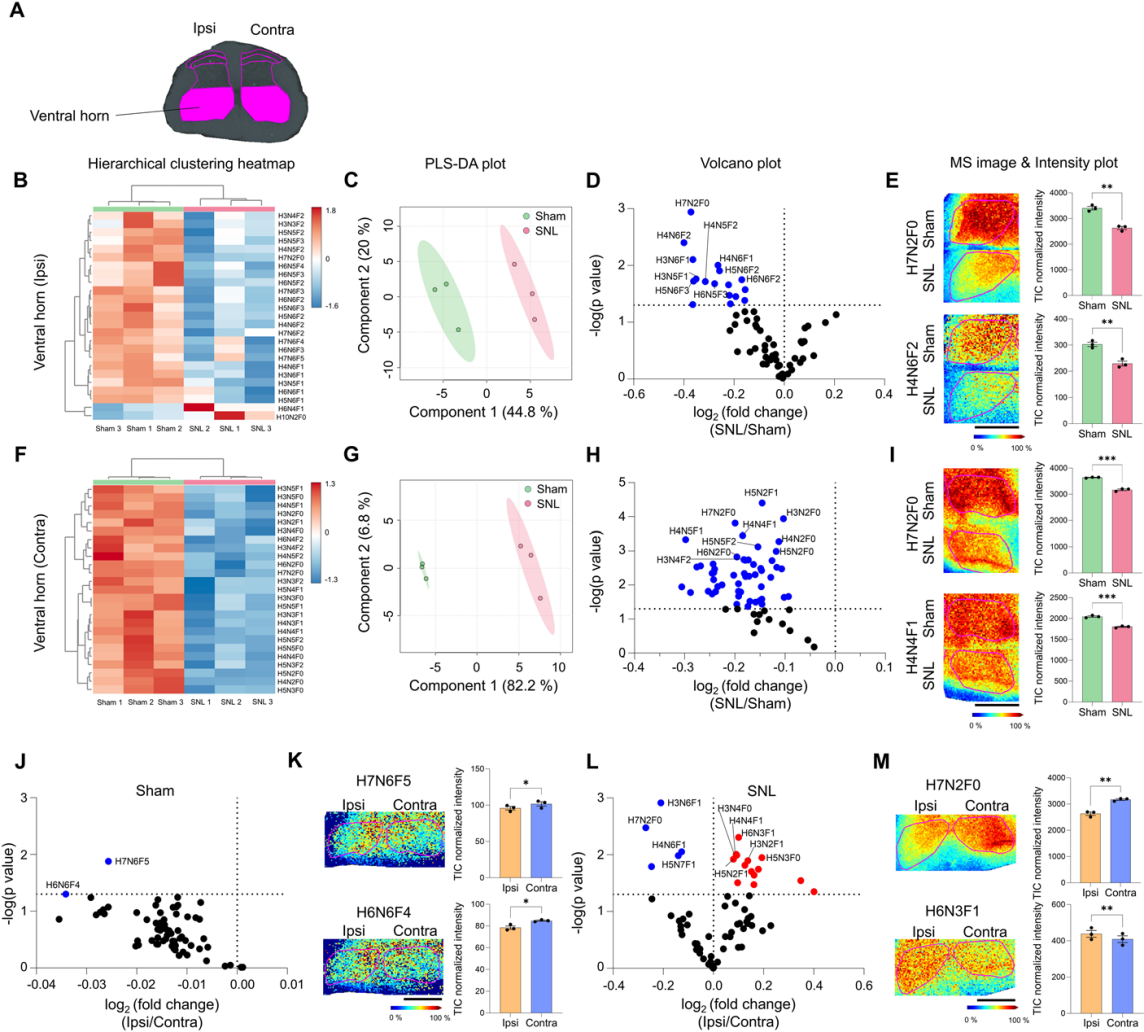
N-glycan expression significantly decreases in the ventral horn of the SNL spinal cord following SNL.** A** ROI of ventral horn. **B**–**I** Hierarchical clustering heatmap, PLS-DA plot, volcano plot, and MALDI mass spectrometry images with intensity plot of the ipsilateral (**B**–**E**) and contralateral (**F**–**I**) ventral horn. The correlation of the top 25 N-glycans detected in three samples from sham (green) and SNL (red) groups is shown and presented in the hierarchical clustering heatmap (H: hexose, N: N-acetylglucosamine, and F: fucose). PLS-DA plot of N-glycans of sham and SNL groups with 95% confidence region. In the volcano plot, blue dots indicated N-glycans significantly decreased in SNL group compared with sham group (*p* < 0.05). Representative MALDI mass spectrometry images and intensity plots of N-glycans reveal significant differences between sham and SNL groups (unpaired *t*-test, **p < 0.01, ***p < 0.001, error bar: SEM). **J**–**M** Volcano plots and MALDI mass spectrometry images with intensity plots of the ipsilateral (Ipsi, orange) and contralateral (Contra, blue) ventral horn of the spinal cord within either sham (**J**, **K**) or SNL (**L**, **M**) groups. In the volcano plots, *p* < 0.05 was considered statistically significant. Representative MALDI mass spectrometry images and intensity plots of N-glycans show significant differences between ipsi and contra sides within each group (paired *t*-test, *p < 0.05, **p < 0.01, error bar: SEM). Data are representative of *n* = 3 independent experiments. Scale bars = 1 mm

The ventral horn, or anterior horn, primarily contains motor neurons that project from the spinal cord to innervate skeletal muscles [40]. Our analysis of N-glycan expression in the ventral horn (Fig. 5A) revealed distinct differences between the sham and SNL groups on both the ipsilateral and contralateral sides, as indicated by heatmap and PLS-DA analyses (Fig. 5B, C, F, G). Consistent with findings in the dorsal horn, most N-glycans were downregulated on both sides of the ventral horn (Fig. 5D, E, H, I and Supplementary Fig. 6B, D). Seventeen and 52 N-glycans were significantly decreased in the ipsilateral and contralateral ventral horns, respectively (Fig. 5D, H). Notably, in the contralateral ventral horn, 52 out of 65 N-glycans showed significant reductions in the SNL group compared with the sham group (Fig. 5H), reflecting substantial changes in this region following neuropathic pain induced by the SNL model.

Additionally, the differential analysis of N-glycan expression between the ipsilateral and contralateral ventral horns within the SNL group showed less prominence in the sham group (Fig. 5J–M and Supplementary Fig. 6E, F), similar to the dorsal laminae IV/V/VI layers. A total of 19 N-glycans exhibited significant differences in the comparison of the ipsilateral and contralateral sides following SNL, with five N-glycans showing a decrease and 14 N-glycans showing an increase (Fig. 5L and Supplementary Table 5). The investigation of lateralization effects within the SNL group revealed that most significant N-glycans were downregulated on the contralateral side, while minimal changes were observed on the ipsilateral side (Supplementary Fig. 7A, B). Again, this implies that the lateralization effects might be due to dynamic changes in the contralateral ventral horn following SNL.

### Neuropathic pain injury induced significant increase in N-glycan expression in thalamus

**Fig. 6:**
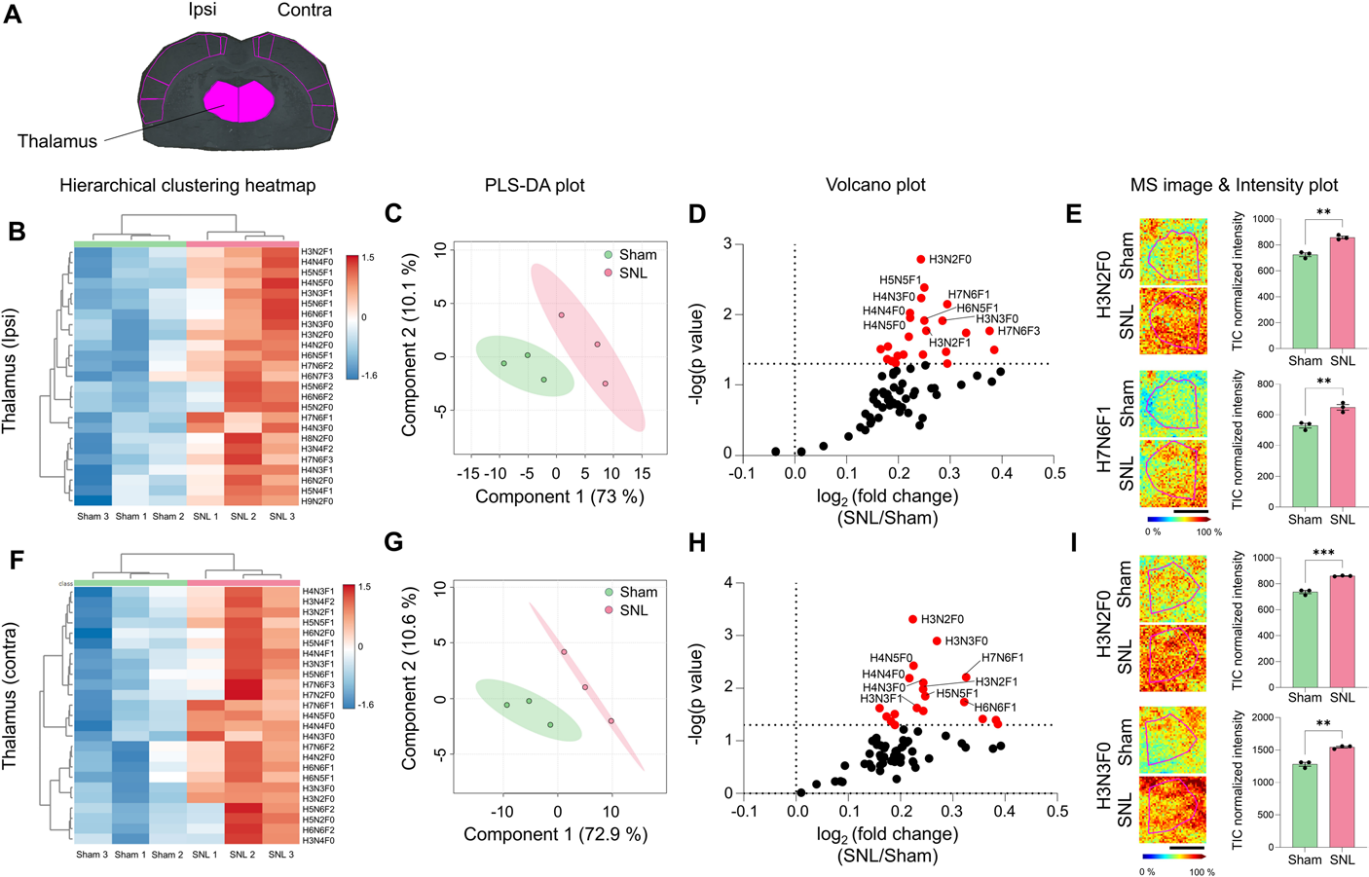
N-glycan expression significantly increases in the thalamus following SNL.** A** ROI of thalamus. **B**–**I** Hierarchical clustering heatmap, PLS-DA plot, volcano plot, and MALDI mass spectrometry images with intensity plot of the ipsilateral (**B**–**E**) or contralateral (**F**–**I**) thalamus. The correlation of the top 25 N-glycans detected in three samples from the sham (green) and SNL (red) groups is shown and presented in the hierarchical clustering heatmap (H: hexose, N: N-acetylglucosamine, and F: fucose). PLS-DA plot of N-glycans of the sham and SNL groups with 95% confidence region. In the volcano plots, *p* < 0.05 was considered statistically significant. Representative MALDI mass spectrometry images and intensity plots of N-glycans show significant differences between the sham and SNL groups (unpaired *t*-test, **p < 0.01, ***p < 0.001, error bar: SEM). Data are representative of *n* = 3 independent experiments. Scale bars = 2 mm

The thalamus serves as the gateway to the cerebral cortex, where nociceptive inputs from the spinal cord converge before reaching the cortex. Through MALDI MSI analysis of brain samples, we detected 72 distinct N-glycans (Supplementary Table 2). To explore changes in N-glycan expression within brain regions involved in pain processing, we focused initially on the thalamus (Fig. 6A). Notably, there was a clear distinction between the sham and SNL groups in N-glycan expression profiles, as revealed by heatmap and PLS-DA analyses (Fig. 6B, C, F, G). Interestingly, in the thalamus, N-glycan expression significantly increased following SNL compared with the sham group, contrasting with the downregulation observed in the spinal cord. This upregulation was evident on both the ipsilateral and contralateral sides of the thalamus, with 23 and 19 N-glycans showing increased expression, respectively (Fig. 6D, E, H, I and Supplementary Fig. 8B, D). Regarding lateralization effects, the baseline sham group exhibited more pronounced lateralized expression of N-glycans, whereas the SNL group showed minimal differences, with only 3 out of 76 N-glycans displaying lateralized expression changes (Supplementary Fig. 8E, F).

### Neuropathic pain injury induced significant increase in N-glycan expression in S1 and S1HL

**Fig. 7:**
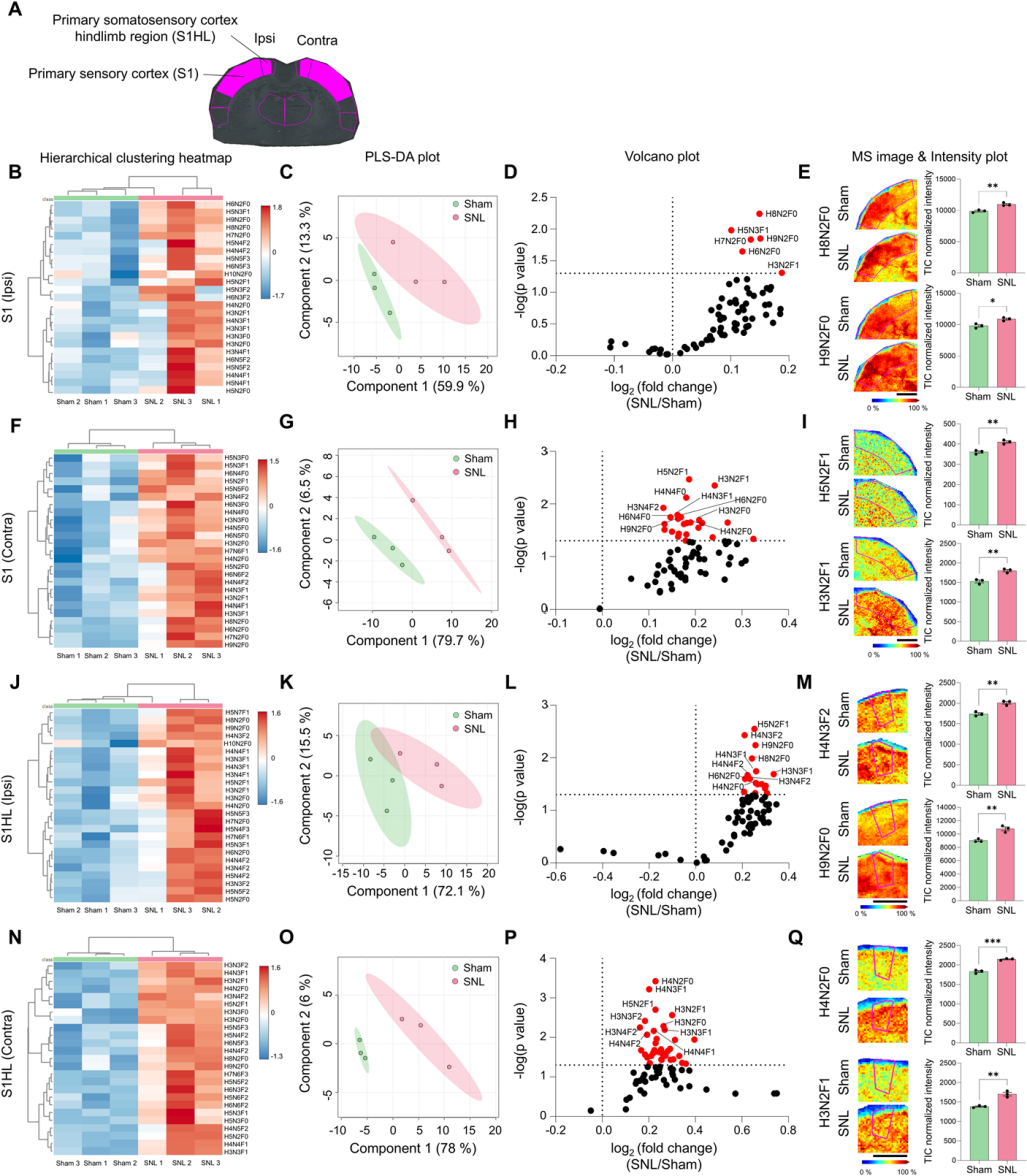
N-glycan expression significantly increases in S1 and S1HL following SNL.** A** ROI of S1 and S1HL. **B**–**Q** Hierarchical clustering heatmap, PLS-DA plot, volcano plot, and MALDI mass spectrometry images with intensity plot of the ipsilateral or contralateral S1 (**B**–**I**) and S1HL (**J**–**Q**). The correlation of the top 25 N-glycans detected in three samples from the sham (green) and SNL (red) groups is shown and presented in the hierarchical clustering heatmap (H: hexose, N: N-acetylglucosamine, and F: fucose). The PLS-DA plot of N-glycans of the sham and SNL groups with 95% confidence region. In the volcano plots, *p* < 0.05 was considered statistically significant. Representative MALDI mass spectrometry images and intensity plots of N-glycans show significant differences between sham and SNL groups (unpaired *t*-test, *p < 0.05, **p < 0.01, ***p < 0.001, error bar: SEM). Data are representative of *n* = 3 independent experiments. Scale bars = 2 mm. The ROI of S1HL is included in the S1 area

The primary somatosensory cortex (S1; Fig. 7A) is a central hub for processing somatosensory information, with substantial evidence supporting its prominent and highly modulated role in the sensory aspects of pain. Interestingly, heatmap and PLS-DA analyses revealed distinct clustering only on the contralateral side of S1, which receives input from the ipsilateral dorsal horn due to the decussation of afferent axons at the spinal cord level (Fig. 7F, G). In contrast, the ipsilateral side of these brain regions did not exhibit clear clustering (Fig. 7B, C). Furthermore, the volcano plot indicated more pronounced alterations in N-glycan expression on the contralateral side of S1 after SNL compared with the sham group (Fig. 7D, H). Specifically, six N-glycans in ipsilateral S1 and 24 N-glycans in contralateral S1 showed significant differences when comparing the sham and SNL groups (Fig. 7D, E, H, I and Supplementary Fig. 9B, D). The changes in N-glycans in the S1HL region were similar to the S1 region, with clustering between groups being clearer in contralateral S1HL than in ipsilateral S1HL (Fig. 7J, K, N, O). In addition, in the comparison of sham and SNL in volcano plot, 17 and 36 N-glycans showed significant differences in ipsilateral and contralateral, respectively (Fig. 7L, M, P, Q and Supplementary Fig. 10B, D). The number of N-glycans showing significant changes observed in S1HL was higher than that in S1 (Supplementary Fig. 9E, F). In S1HL, neither sham nor SNL showed significant differences (Supplementary Fig. 10E, F). This indicated that both hemispheres had similar levels of N-glycans following SNL, with greater biological consistency observed only in the contralateral side of S1 and S1HL.

We investigated N-glycan expression in the secondary sensory cortex (S2, Supplementary Fig. 11A), which is known to be related to the pain matrix. N-glycans in S2 showed clear clustering between the two groups in ipsilateral (Supplementary Fig. 11B, C) but not in contralateral side (Supplementary Fig. 11G, H). The number of N-glycans that showed significant differences in SNL compared with sham was ten in ipsilateral S2 and two in contralateral S2 (Supplementary Fig. 11E, J and Supplementary Table 4). Most N-glycans detected in the S2 region were increased in SNL compared with sham. In addition, there was no significant difference in lateralization within the sham or SNL group, respectively (Supplementary Fig. 11L, M).

Among the spinal cord regions, we observed the most dynamic N-glycan expression changes in the ventral horn (Fig. 5). On this basis, we further investigated the N-glycan expression changes in the primary motor cortex (M1), a brain region that controls motor activities. In the case of M1 (Supplementary Fig. 12A), although the clustering between the two groups of sham and SNL in the ipsilateral M1 was not clear (Supplementary Fig. 12B), the PLS-DA plot distinguished the two groups (Supplementary Fig. 12C, D). In addition, 13 N-glycans that showed significant intensity differences between the sham and SNL groups were observed (Supplementary Fig. 12E, F). In contrast, in the contralateral M1, clustering and distinction between the two groups, sham and SNL, were clear (Supplementary Fig. 12G, H), and 21 N-glycans with significant intensity differences were observed between the two groups, sham and SNL (Supplementary Fig. 12 J, K). The lateralization of each group, sham and SNL, did not show a significant difference (Supplementary Fig. 12L, M).

### Neuropathic pain injury induced significant alterations in N-glycan profiles in the mPFC, ACC, and IC

**Fig. 8:**
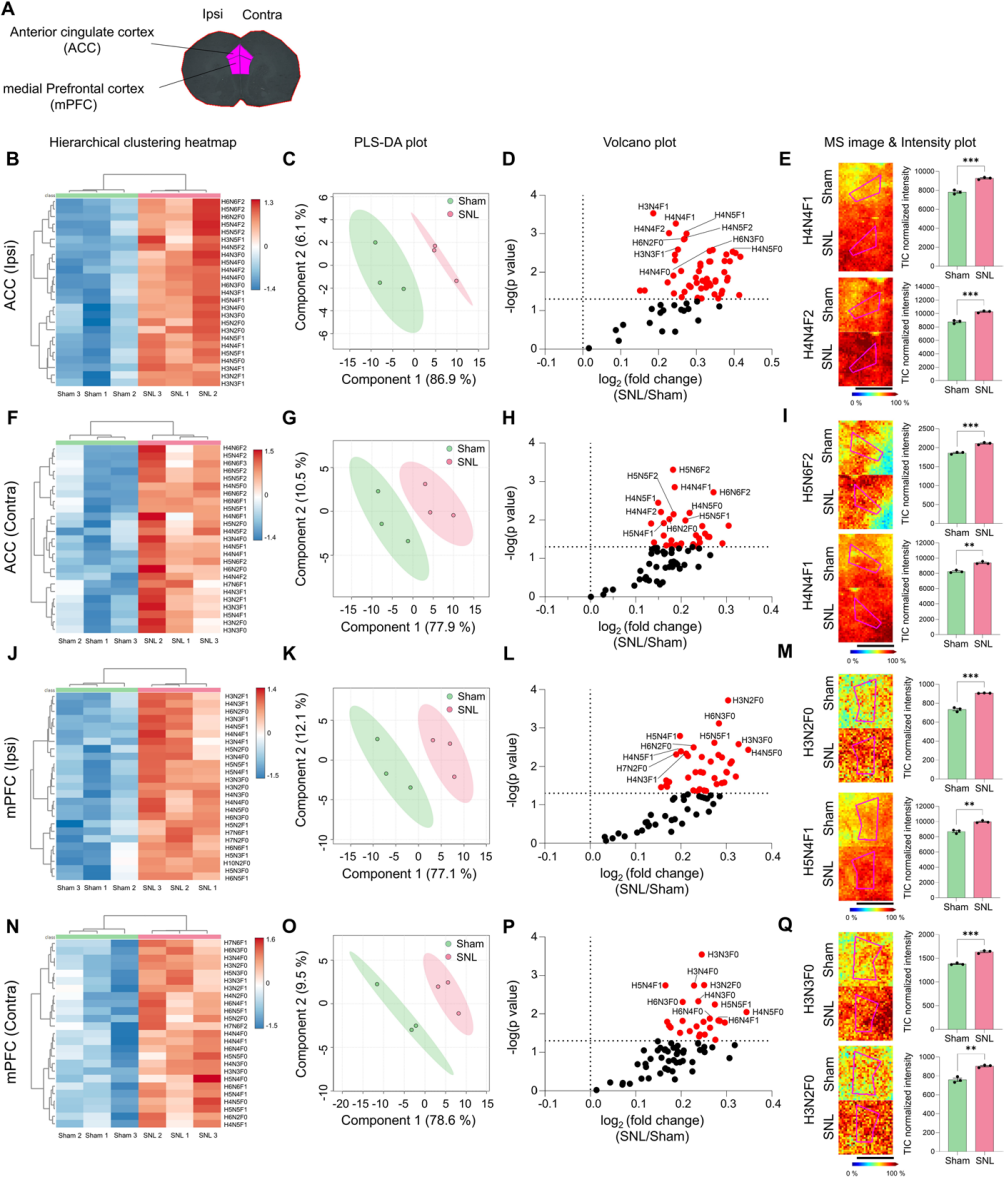
N-glycan expression significantly increases in the ACC and mPFC following SNL. **A** ROI of ACC and mPFC. **B**–**Q** Hierarchical clustering heatmap, PLS-DA plot, volcano plot, and MALDI mass spectrometry images with intensity plot of the ipsilateral or contralateral ACC (**B**–**I**) and mPFC (**J**–**Q**). The correlation of the top 25 N-glycans detected in three samples from the sham (green) and SNL (red) groups is shown and presented in the hierarchical clustering heatmap (H: hexose, N: N-acetylglucosamine, and F: fucose). The PLS-DA plot of N-glycans from the sham and SNL groups with 95% confidence region. In the volcano plots, *p* < 0.05 was considered statistically significant. Representative MALDI mass spectrometry images and intensity plots of N-glycans show significant differences between the sham and SNL groups (unpaired *t*-test, **p < 0.01, ***p < 0.001, error bar: SEM). Data are representative of *n* = 3 independent experiments. Scale bars = 2 mm

Next, we examined brain regions associated with emotional pain, such as the ACC, mPFC, and IC. In the ipsilateral ACC (Fig. 8A), sham and SNL were clearly separated (Fig. 8B, C). Fifty-three N-glycans showed a significant increase in the ipsilateral ACC in SNL compared with sham (Fig. 8D, E, and Supplementary Fig. 13B). Similarly, in the contralateral ACC, the two groups were clearly separated (Fig. 8F, G), and 30 N-glycans showed a significant difference in SNL compared with sham (Fig. 8H, I and Supplementary Fig. 13D). In the sham ACC, a large number of N-glycans were increased in contralateral ACC compared with ipsilateral ACC (Supplementary Fig. 13E and Supplementary Table 5). However, no lateralization was found in SNL ACC (Supplementary Fig. 13F).

The N-glycan expression pattern in the mPFC was similar to that in the ACC. Clear differences between the two groups were observed in both the ipsilateral and contralateral mPFC (Fig. 8J, K, N, O), with 38 and 25 N-glycans showing significant increases, respectively (Fig. 8L, M, P, Q and Supplementary Fig. 14B, D). Lateralization between the sham and SNL groups did not show significant differences (Supplementary Fig. 14E, F). Again, most N-glycans detected in the ACC and mPFC exhibited increased relative intensities in the SNL group compared with the sham group (Fig. 8D, H, L, P).

In the case of the IC (Supplementary Fig. 15A), the N-glycans detected in the ipsilateral and contralateral IC did not clearly cluster between the two groups (Supplementary Fig. 15B, C, F, G), and no N-glycans showing significant differences were observed (Supplementary Fig. 15E, I). In addition, two N-glycans contributed to lateralization in sham, but were not found in SNL (Supplementary Fig. 15 J, K). These results suggest that N-glycan expression alterations are not uniform across the brain but are specific to certain regions.

## Discussion

**Fig. 9:**
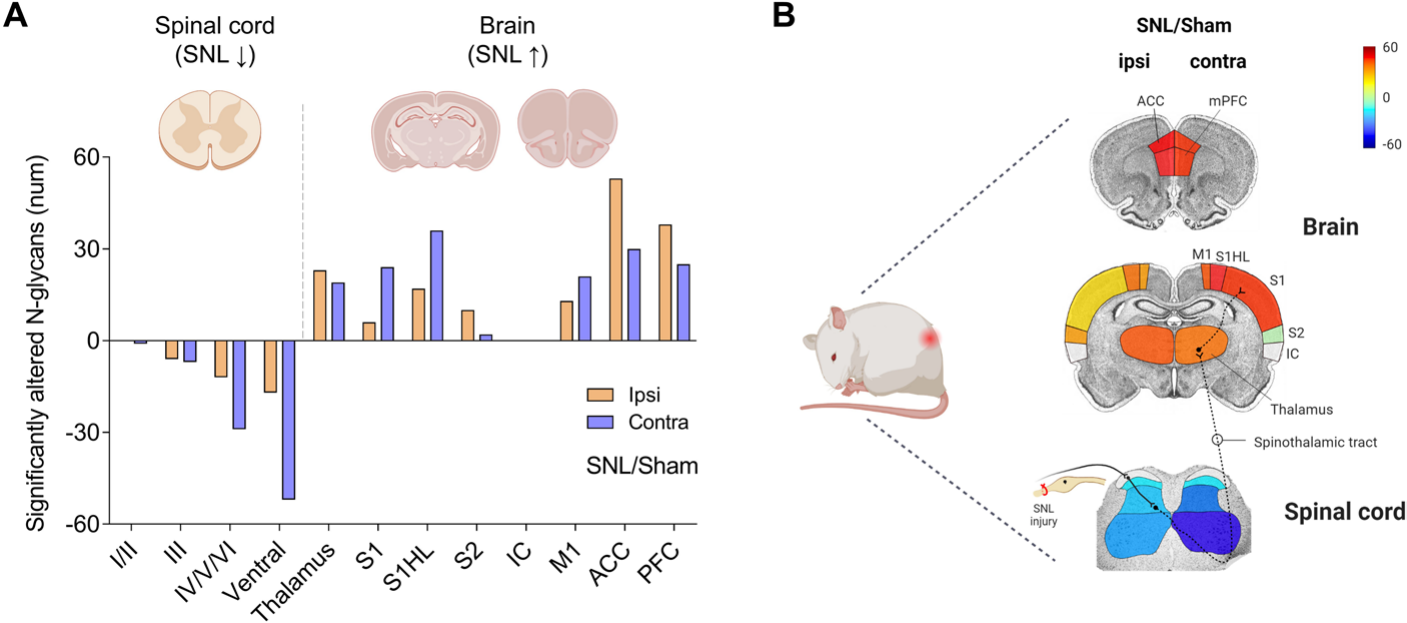
Graphical summary of significantly altered N-glycans in the spinal cord and brain following SNL. **A** N-glycan expression was significantly decreased in the spinal cord and increased in the brain of the SNL group compared with the sham group. **B** The color-coded image represents the number of significantly altered N-glycans, as shown in the bar graph, visualizing changes in each ROI following SNL injury

This study is the first to simultaneously examine N-glycan dynamics in both the spinal cord and multiple brain regions following spinal nerve ligation (SNL), a neuropathic pain model. The results demonstrate that neuropathic pain stimuli and/or intracranial plasticity induced by such stimuli induces region-specific N-glycosylation changes, with notable bidirectional alterations between the spinal cord and brain. Interestingly, lateralization effects in N-glycan expression were more prominent in the spinal cord than in the brain, suggesting distinct molecular mechanisms in these regions. Utilizing the powerful MALDI MSI technique, this study successfully reveals how N-glycosylation varies across the central nervous system during neuropathic pain (Fig. 9). These findings provide new insights into N-glycan function, paving the way for future research and the development of novel biomarkers and targeted therapeutic strategies for neuropathic pain.

### Neuroinflammation and N-glycosylation in spinal cord following SNL

Our results show that neuropathic pain is closely associated with robust glial activation and heightened neuroinflammation in the ipsilateral dorsal and ventral horns of the spinal cord. Using the SNL model, a well-established method for inducing neuropathic pain, we observed a significant increase in pro-inflammatory mediators, including TNF-α, IL-1β, IL-6, and iNOS, on day 14 of injury. These findings are consistent with previous studies that emphasize the role of glia in mediating pain hypersensitivity through the release of pro-inflammatory cytokines, contributing to the persistence of neuropathic pain [41]. Thus, our model provides a reliable framework for investigating N-glycan expression in the context of neuropathic pain.

Despite the well-established role of superficial laminae (I/II) and lamina III in relaying nociceptive Aδ and C fiber input in the dorsal horn, we did not observe significant changes in N-glycan expression in these areas following SNL, which was unexpected. This is particularly surprising given the clear glial activation in laminae I/II/III on the ipsilateral side of the spinal cord in our model. Recent studies suggest that N-glycosylation is more closely linked to neurons than glia, as indicated by the predominance of N-glycan expression in gray matter compared with white matter [42]. Interestingly, we found a stronger distribution of c-Fos-positive neurons in the deep dorsal horn than in the superficial layer, and similarly, N-glycan expression changes were more pronounced in the deep layers of the spinal cord than in the superficial layers. This suggests a potential correlation between N-glycan expression and neuronal activation in spinal cord in response to neuropathic pain. However, it remains unclear why N-glycan expression exhibits more pronounced changes in the contralateral laminae IV/V/VI and ventral horn, despite the injury being ipsilateral. c-Fos staining did not reveal significant differences in the number of active neurons between hemispheres, likely because N-glycan expression was analyzed at 2 weeks when pain persists, and c-Fos, an immediate early gene, reflects short-term neuronal activity. Additionally, given that rats tend to shift weight to the contralateral hindlimb to compensate for pain in the injured limb—and considering that laminae IV/V/VI relay non-nociceptive input and the ventral horn controls motor function—these changes may represent an adaptive response to regulate movement and sensation in the contralateral hindlimb. In addition, while MALDI-MSI cannot resolve N-glycan alterations at the cell-type level, complementary techniques such as single-cell RNA sequencing and studies on enzymes involved in N-glycan biosynthesis could provide a deeper understanding of cell type-specific modulation of N-glycosylation in response to pain. These approaches could identify key enzymes and their associated N-glycan biosynthetic pathways, which drive changes in specific N-glycan types. This knowledge could enable targeted modulation of N-glycosylation in particular regions and cell populations of the spinal cord or brain, offering a promising strategy for managing neuropathic pain. Further investigation into the cellular sources of these N-glycosylation changes could shed light on the molecular mechanisms underlying neuropathic pain.

### N-glycosylation in brains following SNL

Notably, alterations in N-glycan expression within the primary sensory cortex (S1) were more pronounced in the contralateral hemisphere. We identified six significant N-glycans in the ipsilateral S1, compared with 25 in the contralateral S1, suggesting more dynamic shifts in N-glycosylation on the contralateral side. This observation aligns with the decussation of sensory pathways at the spinal cord, reinforcing the persistence of lateralization in this brain region. Moreover, the heatmap and PLS-DA plot showed weaker clustering in the ipsilateral S1, indicating reduced biological consistency in this region. By contrast, brain regions such as the ACC and mPFC from the same animals demonstrated clear clustering, even on the ipsilateral side. Thus, the distinct alterations in N-glycan expression are particularly evident in the contralateral S1, including S1HL. These findings emphasize the need for future studies to pinpoint upstream modulators and downstream targets, which will be pivotal in elucidating the lateralized modulation of N-glycosylation in neuropathic pain within the primary sensory cortex.

The differential expression observed in the ACC and mPFC in the SNL group suggests that N-glycosylation changes are involved in both the sensory and emotional aspects of pain processing. Interestingly, lateralization in these emotional brain regions was less pronounced, indicating that the emotional processing of pain may be more generalized across both hemispheres. This suggests that, while sensory processing retains some lateralization, the emotional response to pain engages both hemispheres more equally. A recent study shows that pain perception is processed by various brain networks, with higher-order networks such as the limbic system and default mode network integrating both expectations and actual sensory information to reconstruct the pain experience [43]. Additionally, the study also shows that participants who anticipated greater pain reported feeling more pain, even with the same stimulus intensity, suggesting that both pain expectations and the actual stimulus combine to determine the perceived pain. Therefore, consistent with these recent findings, our study suggests that brain-related changes are more pronounced than spinal cord alterations, with N-glycan modifications potentially serving as key molecular mediators. The observed glycosylation changes are early molecular events that may contribute to the delayed onset of emotional processing alterations over time, potentially through mechanisms such as synaptic or circuit-level plasticity [44]. Future studies combining glycoproteomics with longitudinal behavioral assessments and interventions targeting glycosylation-related enzymes (e.g., glycosyltransferases) could help elucidate these mechanisms and establish causal links between glycosylation changes in ACC and mPFC and anxiety-like behaviors.

We did not observe significant changes in N-glycan profiles in the IC, a key region involved in processing emotional and motivational aspects of pain [45, 46]. This lack of change may be because the IC is more closely associated with acute or immediate pain, rather than sustained neuropathic pain [47]. Some studies suggest that, in persistent pain states, the role of the IC diminishes as the pain experience becomes more generalized and less localized [48]. While the responsiveness of the IC to immune modulation in the acute phase has been noted [49, 50], it is controversial whether the IC plays a more significant role during the acute or chronic phase. Studies have shown that, when the plastic changes in the IC are inhibited by blocking glutamatergic transmission, analgesic effects are observed in neuropathic pain models, suggesting potential involvement in chronic pain processing [51–53]. Thus, further research will be necessary to explore the role of N-glycosylation in acute versus chronic pain.

### Bidirectional changes in N-glycan expression between the spinal cord and brain

We observed a notable and significant bidirectional alteration in N-glycans between the spinal cord and the brain on day 14 following SNL (Fig. 9). In the spinal cord, N-glycan expression was significantly downregulated. This trend toward decreased N-glycan levels was evident even in regions where the changes were not statistically significant. Conversely, in key brain regions involved in sensory and emotional pain processing—such as the thalamus, primary sensory cortex (SI and S1HL), anterior cingulate cortex (ACC), and medial prefrontal cortex (mPFC)—N-glycan expression was significantly upregulated. This upward trend was also observed in regions such as the S2 and IC, despite the changes not reaching statistical significance. These findings indicate that SNL induces a marked downregulation of N-glycosylation in the spinal cord, resulting in fewer N-glycans attached to glycoproteins. Conversely, there is a significant upregulation of N-glycosylation in the brain, leading to an increase in N-glycans on glycoproteins. This is measured by our method, which uses PNGase F to assess N-glycans attached to asparagine residues on glycoproteins. The contrasting patterns of N-glycosylation response in the spinal cord and brain suggest that their regulatory mechanisms may differ. In the future, it would be significant to investigate whether N-glycan expression changes exhibit dynamic patterns during pain progression over time, particularly in the early and later stages. Additionally, exploring whether interventions targeting these alterations can impact pain outcomes would provide valuable insights.

Identifying the upstream regulators responsible for the differential N-glycosylation changes observed in the spinal cord and brain could yield valuable insights into the mechanisms underlying neuropathic pain processing and modulation. Since the dorsal laminae IV/V/VI of the spinal cord exhibited the most significant changes, and these areas contain neurons that project to the brain, we analyzed N-glycans significantly altered in the SNL model compared with the sham group, from the dorsal laminae IV/V/VI to the thalamus and S1 cortex (Supplementary Fig. 16A). We identified a specific N-glycan structure, H6N6F2, along the spinothalamic tract (Supplementary Fig. 16A, B). This fucosylated N-glycan showed bidirectional expression after SNL, with a decrease in the ipsilateral dorsal laminae IV/V/VI and an increase in both the thalamus and S1 cortex compared with the sham group (Supplementary Fig. 16C). Additionally, although the total number of identified glycans differed between the spinal cord (65) and the brain (76), we observed much less overlap in N-glycan expression between the spinal cord and brain (two shared glycans between the dorsal horn and thalamus, and two between the dorsal horn and S1 cortex) compared with between different brain regions (12 shared glycans between the thalamus and S1 cortex) (Supplementary Fig. 16A). The thalamus is a crucial brain region involved in both sensory and motor processing. Our data reveal distinct N-glycan expression patterns in both the ipsilateral and contralateral thalamus, as shown in the heatmap and PLS-DA plot. Since pain signals cross at the spinal cord and are relayed to the contralateral side of the brain, the alterations observed in the contralateral thalamus and primary motor cortex (M1) are likely due to pain sensory input and associated motor changes. An intriguing finding, however, is the significant alteration in N-glycan expression in the ipsilateral thalamus and M1. While the heatmap for the ipsilateral M1 does not show as clear clustering as seen in the contralateral M1, the PLS-DA plot clearly distinguishes between the sham and SNL groups. Additionally, numerous N-glycan changes were observed in the ipsilateral M1, suggesting that N-glycosylation alterations in both the ipsilateral thalamus and M1 may contribute to motor adaptations resulting from pain-induced compensatory behaviors. More pronounced changes were seen in the contralateral dorsal laminae IV/V/VI layers and the contralateral ventral horn. These alterations can be explained by the tendency of rats to shift weight to the contralateral hindlimb to compensate for pain in the injured limb. The dorsal laminae IV/V/VI layers relay non-nociceptive input, which subsequently signals to the ipsilateral thalamus and M1, then sends signals to regulate the contralateral ventral horn. Thus, these changes may represent an adaptive response to regulate sensation and movement in the contralateral hindlimb. Taken together, these findings suggest that N-glycosylation alterations not only respond to sensory input, such as pain, but also likely play a role in modulating motor adaptations. The relevance of N-glycan alterations in circuit activation is unclear; however, given the regulation of N-glycosylation on significant glycoproteins such as ion channels and receptors that modulate synaptic plasticity and transmission [54, 55], identifying the upstream modulators and downstream targets of N-glycosylation could provide novel and valuable insights into the molecular mechanisms underlying neuropathic pain. Collectively, these findings suggest that N-glycosylation may exhibit distinct characteristics between the spinal cord and the brain. Further research focused on identifying upstream modulators and downstream target glycoproteins in these regions could clarify how N-glycosylation influences neuropathic pain across the central nervous system.

This study has several limitations that should be acknowledged. First, in this study, only male animals were used to minimize potential variability associated with hormonal cycles in females [56]. However, we recognize that this approach limits the generalizability of our findings. Future studies should include both male and female animals to comprehensively investigate potential sex-specific differences in N-glycosylation and its role in the response to pain. Second, our study focused on a single time point, limiting the ability to fully understand N-glycan alterations and their role in pain signaling. Future investigations should include multiple time points, encompassing both acute pain phases and long-term observations beyond 2 months, to better capture the dynamic changes in N-glycosylation during pain development and persistence. Third, while this study primarily focused on alterations in N-glycan expression within the central nervous system, future research could benefit from exploring the peripheral nervous system, including the dorsal root ganglion, to better elucidate the relationship between N-glycosylation changes and the systemic processing of neuropathic pain. Lastly, we utilized a small number of experimental animals for MALDI MSI. While many previous mass spectrometry imaging studies, including ours, have employed the minimum number of animals necessary for statistical analysis [14, 57–60], we acknowledge that future studies with a larger sample size could enable more robust statistical analyses. Additionally, such studies may reveal subtle patterns in N-glycan distribution across the spinal cord and various brain regions.

Our findings provide valuable insights into regional changes in N-glycan expression, though the functional relevance of specific N-glycan structures remains to be fully elucidated. The most important finding of our study was that, 14 days after neuropathic pain, the expression intensity of various N-glycans significantly decreased in the spinal cord but significantly increased in brain regions. This intriguing result suggests that, despite both being part of the central nervous system, N-glycosylation changes exhibit opposing patterns in the spinal cord and the brain in response to pain. However, it remains challenging to understand how specific glycans functionally contribute to pain processing. To explore this further, we categorized the detected N-glycans into four structural types (OM-F0, OM-F1, C/H-F0, and C/H-F) and analyzed their relative abundance between sham and SNL groups (Supplementary Fig. 17). Notable alterations included a decrease in C/H-F N-glycans (complex/hybrid N-glycans with fucose) in the ipsilateral dorsal laminae IV/V/VI layers, as well as in the contralateral ventral horn, ipsilateral ACC, ipsilateral mPFC, and contralateral mPFC (Supplementary Fig. 17E, H, O, Q, R). Additionally, there was an increase in OM-F0 N-glycans (oligomannose-type N-glycans) in the ipsilateral S1 and contralateral ACC (Supplementary Fig. 17 K, P). These findings suggest that specific N-glycan biosynthetic pathways in certain spinal cord or brain regions may play a significant role in modulating N-glycan expression profiles. Further glycoproteomic analysis could identify glycoproteins and their glycosylation sites, shedding light on alterations in complex/hybrid or oligomannose-type N-glycans in response to neuropathic pain. Such insights may reveal novel protein targets for therapeutic intervention. In addition, while MALDI-MSI cannot resolve N-glycan alterations at the cell-type level, complementary techniques such as single-cell RNA sequencing and studies on N-glycan biosynthetic enzymes could provide a deeper understanding of cell type-specific modulation of N-glycosylation in response to pain. These approaches may identify key enzymes and biosynthetic pathways driving changes in specific N-glycan types, enabling targeted modulation of N-glycosylation in particular spinal cord and brain regions and cell populations. This strategy holds promise for managing neuropathic pain. Although this study does not establish the direct functional relevance of N-glycan alterations in neuropathic pain processing, it is worth emphasizing that it provides the first evidence of opposing N-glycan regulation in the spinal cord and brain within the same animal model following neuropathic pain. These findings suggest a potential role of N-glycosylation in neuropathic pain and lay the groundwork for future research to elucidate its precise mechanisms.

## Conclusions

Overall, our findings suggest that neuropathic pain induces complex, region-specific alterations in N-glycosylation throughout the CNS. The differential patterns of N-glycan expression in the spinal cord, thalamus, sensory cortex, and pain-related emotional brain regions indicate a potential involvement of N-glycosylation in various aspects of pain processing.

## Supplementary Information

Supplementary file 1.

Supplementary file 2.

## Data Availability

The data that support the findings of this study are available from the corresponding author upon reasonable request. Some parts of the graphical abstract, as well as Fig. 1 and 9, were created with BioRender (https://www.biorender.com).

## Abbreviations

MALDI MSI: Matrix-assisted laser desorption/ionization mass spectrometry imaging
S1: Primary sensory cortex
S1HL: Primary sensory cortex hindlimb
S2: Secondary sensory cortex
M1: Primary motor cortex
ACC: Anterior cingulate cortex
mPFC: Medial prefrontal cortex
GFAP: Glial fibrillary acidic protein
Iba1: Ionized calcium-binding adaptor molecule 1
TIC: Total ion count
TNF- α: Tumor necrosis factor-α
IL-1β: Interleukin-1β
IL-6: Interleukin-6
IL-4: Interleukin-4
iNOS: Inducible nitric oxide synthase
POD: Postoperative day
PLS-DA: Partial least squares-discriminant analysis
VIP: Variable importance in projection
