# Supplementary material for: Deciphering distinct spatial alterations in N-glycan expression profiles in the spinal cord and brain of male rats in a neuropathic pain model
Source: Cell Mol Biol Lett. 2025 Mar 11;30:31. doi: 10.1186/s11658-025-00709-7 (PMC11895249; doi:10.1186/s11658-025-00709-7)
Supplement: Supplementary file 1 [file 11658_2025_709_MOESM1_ESM.docx]

**Supplementary Information for**

**Deciphering distinct spatial alterations in N-glycan expression profiles in the spinal cord and brains in a rat model of neuropathic pain**

Hyun Jun Jang^1,*^, Juhee Shin^1,*^, Sangkyu Lee^1^, Boyoung Lee^2,#^, Dong Woon Kim^3,#^

^1^Center for Cognition and Sociality, Institute for Basic Science, Daejeon, Republic of Korea.

^2^Center for Cognition and Sociality, Institute for Basic Science, Daejeon, Republic of Korea. blee@ibs.re.kr

^3^Department of Oral Anatomy & Developmental Biology, Kyung Hee University College of Dentistry, Seoul, Republic of Korea. visnu528@khu.ac.kr

* These authors contributed equally

#Corresponding authors

Boyoung Lee, Email: blee@ibs.re.kr

Dong Woon Kim, Email: visnu528@khu.ac.kr


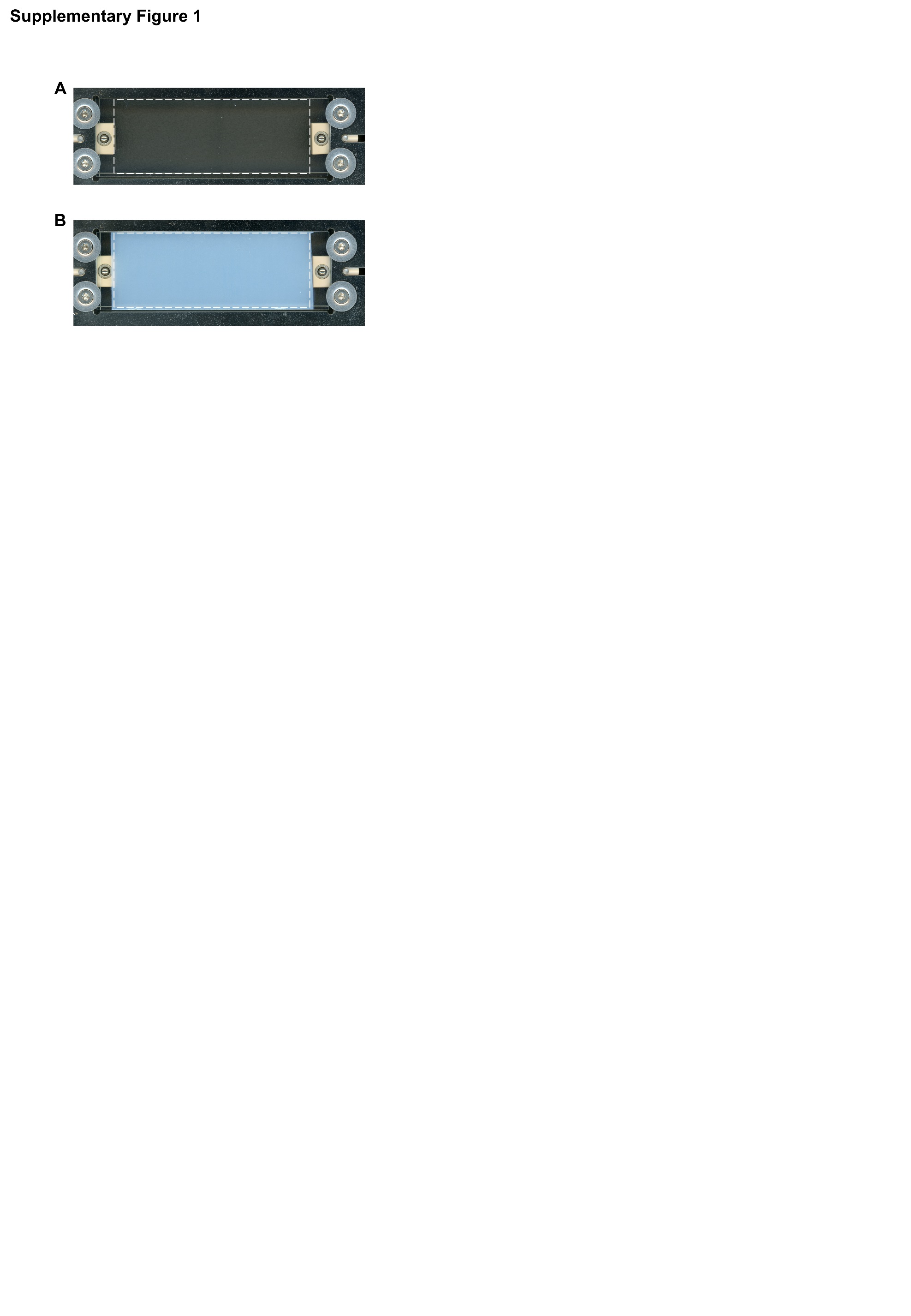


**Supplementary Figure. 1** Optical image of an ITO slide sprayed with CHCA using an M3+ sprayer. The uniform spraying performance of the sprayer used for applying the enzyme and matrix was evaluated by comparing the empty ITO slide (A) and the ITO slide sprayed with the CHCA matrix (B). The white dashed line indicates the area where the CHCA matrix has been sprayed

**
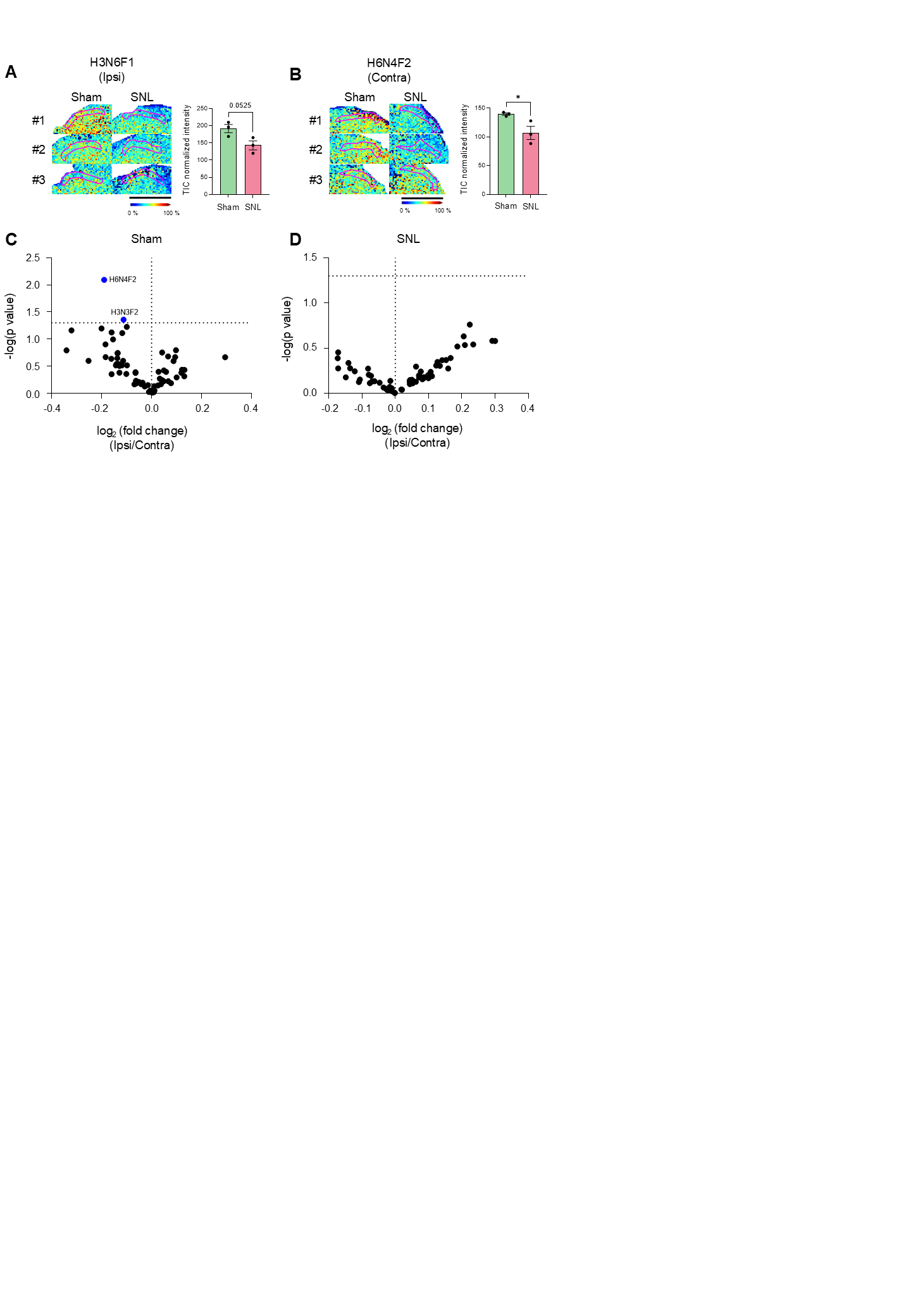
**

**Supplementary Figure. 2** N-glycan expression changes in the dorsal laminae I/II layers of the spinal cord following SNL. **A-B** MALDI mass spectrometry images of all three samples analyzed from Fig. 1e and Fig. 1i, with H3N6F1 for the ipsilateral (**A**) and H6N4F2 for the contralateral (**B**) dorsal laminae I/II layers (unpaired t-test, **p*<0.05, error bars: SEM). H: Hexose, N: N-acetylglucosamine, F: Fucose. Scale bars = 1 mm. **C-D** Volcano plots comparing ipsilateral and contralateral dorsal laminae I/II layers in sham (**C**) and SNL (**D**) groups. *p*<0.05 was considered statistically significant.  Data are representative of *n*=3 independent experiments


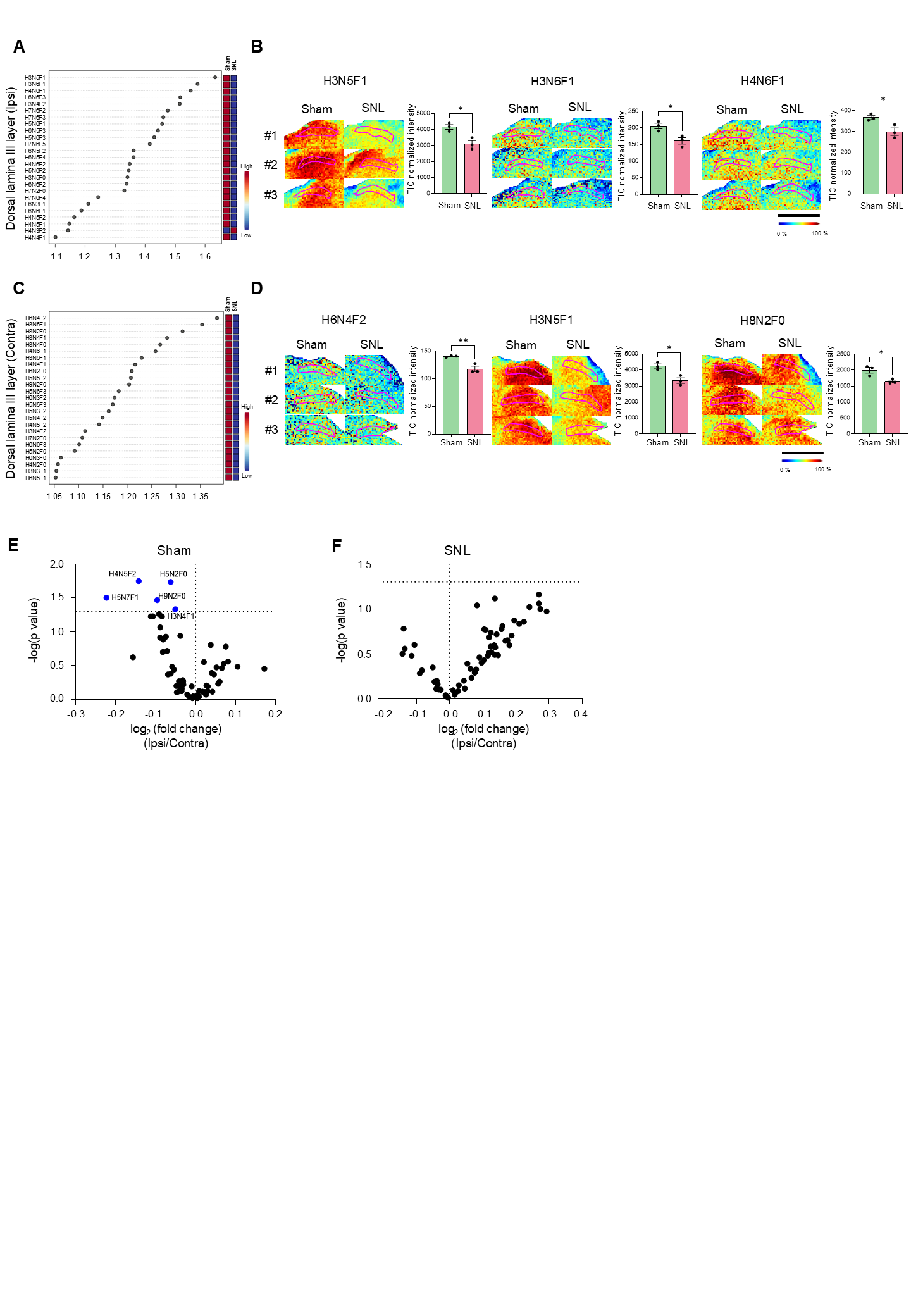


**Supplementary figure. 3** N-glycan expression changes in the dorsal lamina III layer of the spinal cord following SNL. **A-D** Variable importance in projection (VIP) score plot (**A**, **C**) and MALDI mass spectrometry images of all three samples analyzed from Fig. 1m (**B**) and Fig.1q (**D**), for the ipsilateral (**A**, **B**) and for the contralateral (**C**, **D**) dorsal lamina III layers (unpaired t-test, **p*<0.05, ***p*<0.01, error bars: SEM). H: Hexose, N: N-acetylglucosamine, F: Fucose. Scale bars = 1 mm. **E-F** Volcano plots comparing ipsilateral and contralateral dorsal lamina III layers in sham (**E**) and SNL (**F**) groups. *p*<0.05 was considered statistically significant.  Data are representative of *n*=3 independent experiments

**
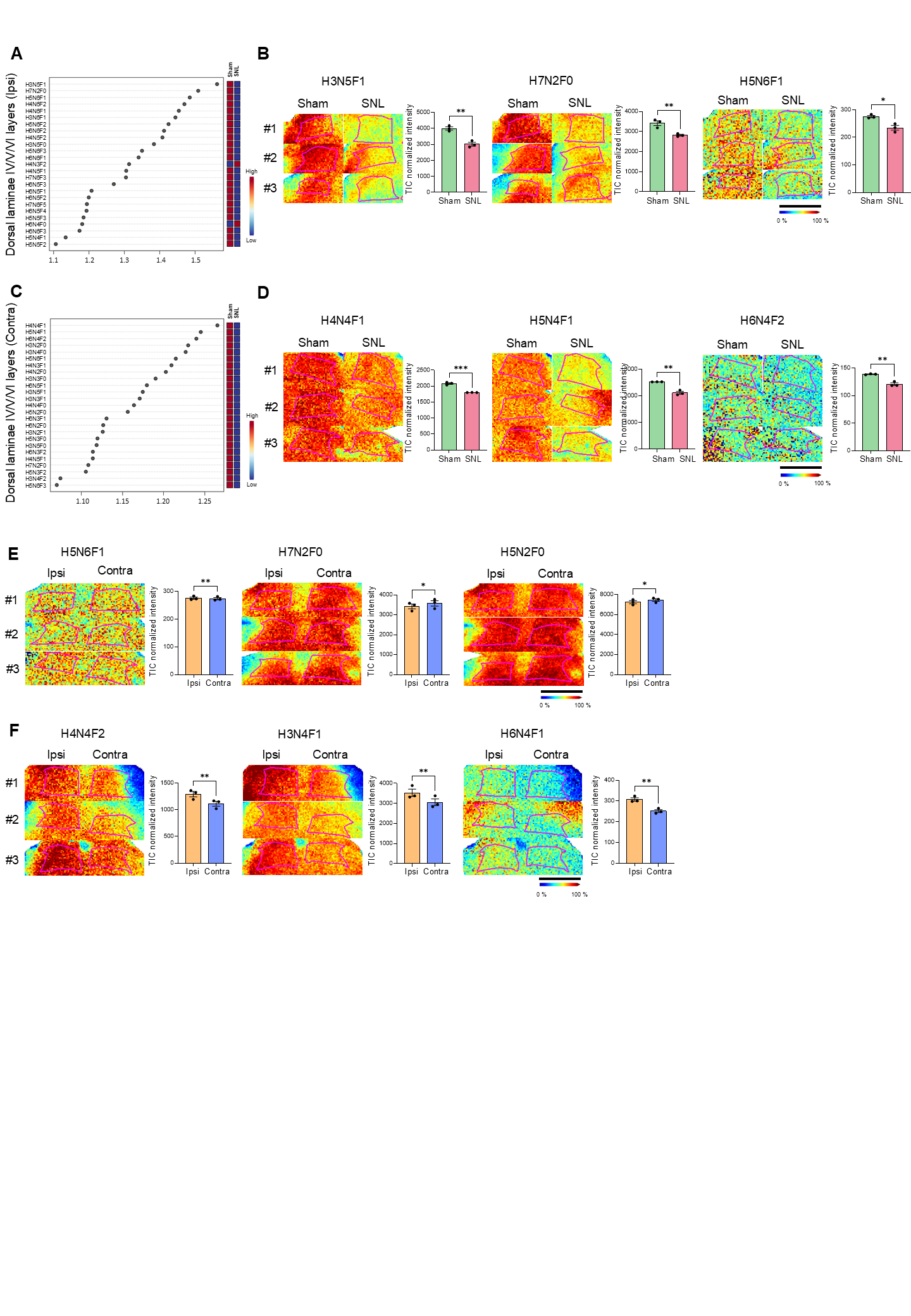
**

**Supplementary figure. 4** Expression changes in N-glycan in the dorsal laminae IV/V/VI layers of the SNL spinal cord. **A-D** Variable importance in projection (VIP) score plot MALDI mass spectrometry images with intensity plot for ipsilateral (**A**, **B**) and contralateral (**C**, **D**) dorsal laminae IV/V/VI layers. The VIP score highlights the top 25 N-glycans that contributed to the differences between the sham and SNL groups in the PLS-DA plot. (unpaired t-test, **p*<0.05, ***p*<0.01, ****p*<0.001, error bar: SEM, H: Hexose, N: N-acetylglucosamine, and F: fucose). **E-F** MALDI mass spectrometry images with intensity plot of the dorsal laminae IV/V/VI layers for sham (**E**) and SNL (**F**). The MS images of the N-glycans with high VIP scores are presented (paired t-test, **p*<0.05, ***p*<0.01, error bar: SEM). Scale bars = 1 mm. Data are representative of *n*=3 independent experiments


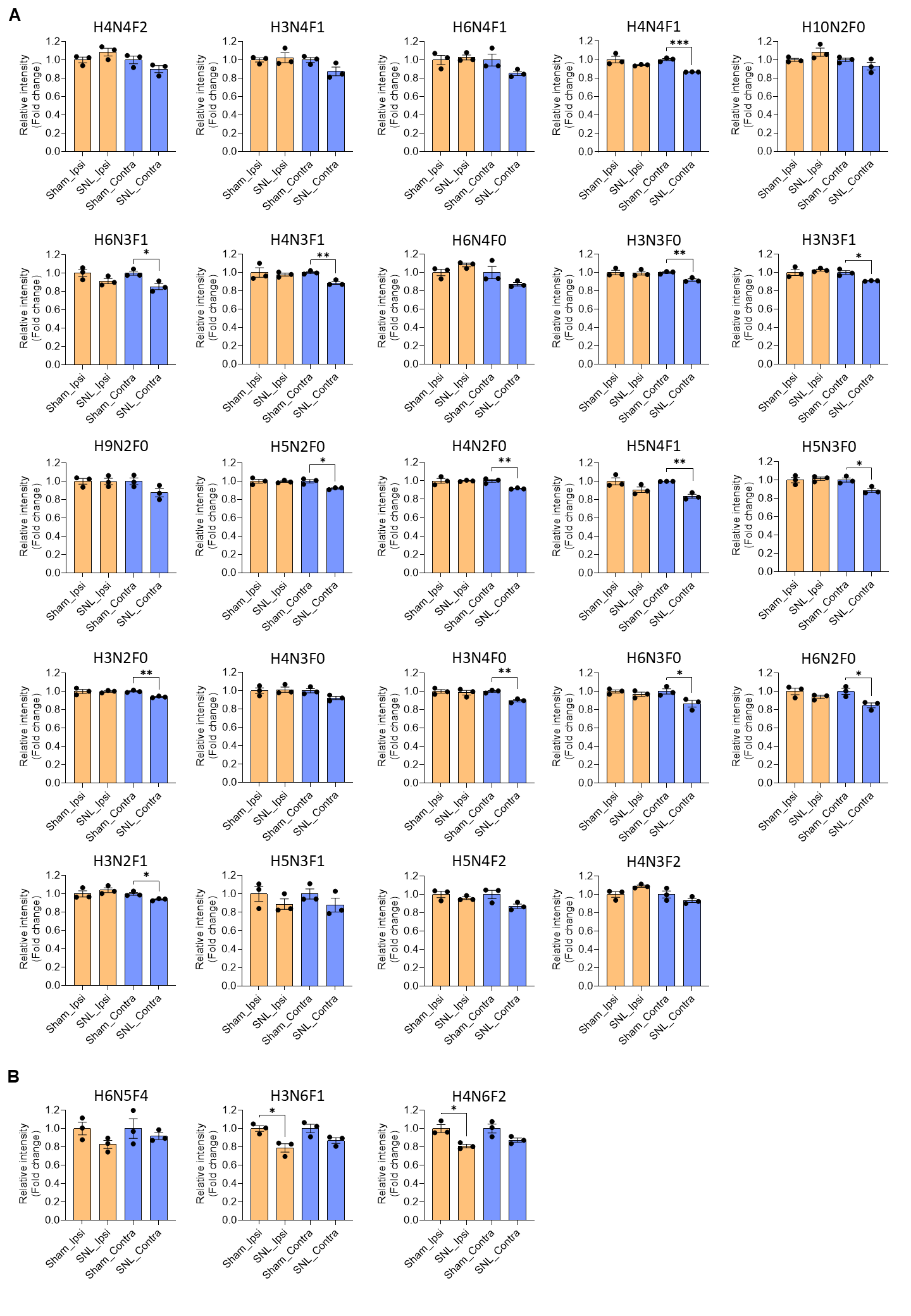


**Supplementary figure. 5** Differential analysis of N-glycan expression between ipsilateral and contralateral dorsal laminae IV/V/VI layers within the SNL group. **A** Twenty-four N-glycans with significantly increased relative intensity in the ipsilateral dorsal laminae IV/V/VI layers of SNL. **B** Three N-glycans with significantly decreased relative intensity in the ipsilateral dorsal laminae IV/V/VI layers of SNL. The value for Ipsilateral and contralateral SNL were expressed as fold change values relative to the ipsilateral and contralateral sham groups, respectively. (unpaired t-test, **p*<0.05, ***p*<0.01, ****p*<0.001, error bar: SEM, H: Hexose, N: N-acetylglucosamine, and F: fucose). Data are representative of *n*=3 independent experiments

**
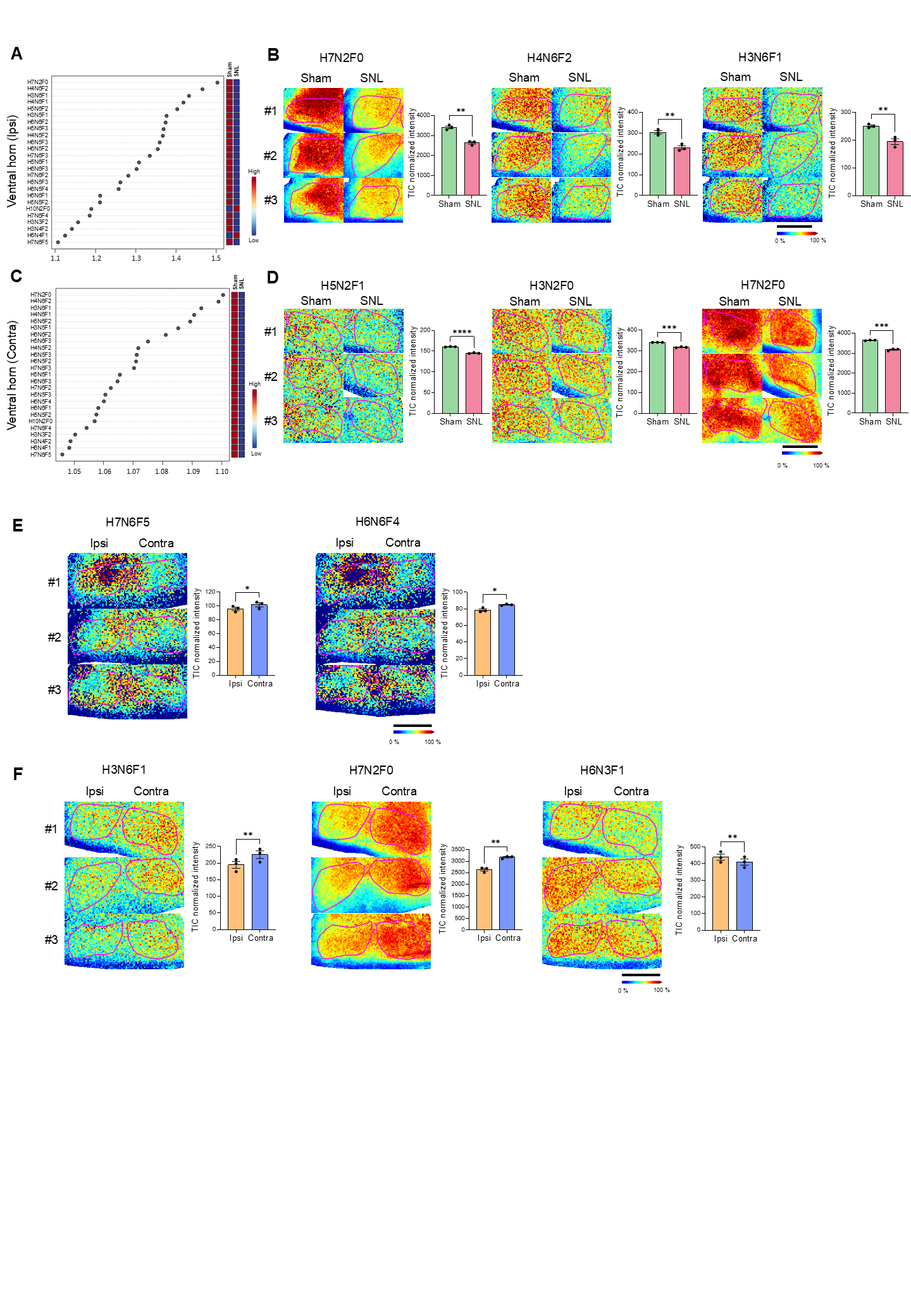
**

**Supplementary figure. 6** Expression changes in N-glycan in the ventral horn of the SNL spinal cord. **A-D** Variable importance in projection (VIP) score plot MALDI mass spectrometry images with intensity plot for ipsilateral (**A**, **B**) and contralateral (**C**, **D**) ventral horn samples. The VIP score highlights the top 25 N-glycans that contributed to the differences between the sham and SNL groups in the PLS-DA plot. (unpaired t-test, ***p*<0.01, ****p*<0.001, *****p*<0.0001, error bar: SEM, H: Hexose, N: N-acetylglucosamine, and F: fucose). **E-F** MALDI mass spectrometry images with intensity plots of the ventral horn for sham (**E**) and SNL (**F**). The mass spectrometry images of the N-glycans with high VIP scores are presented (paired t-test, **p*<0.05, ***p*<0.01, error bar: SEM). Scale bars = 1 mm. Data are representative of *n*=3 independent experiments

**
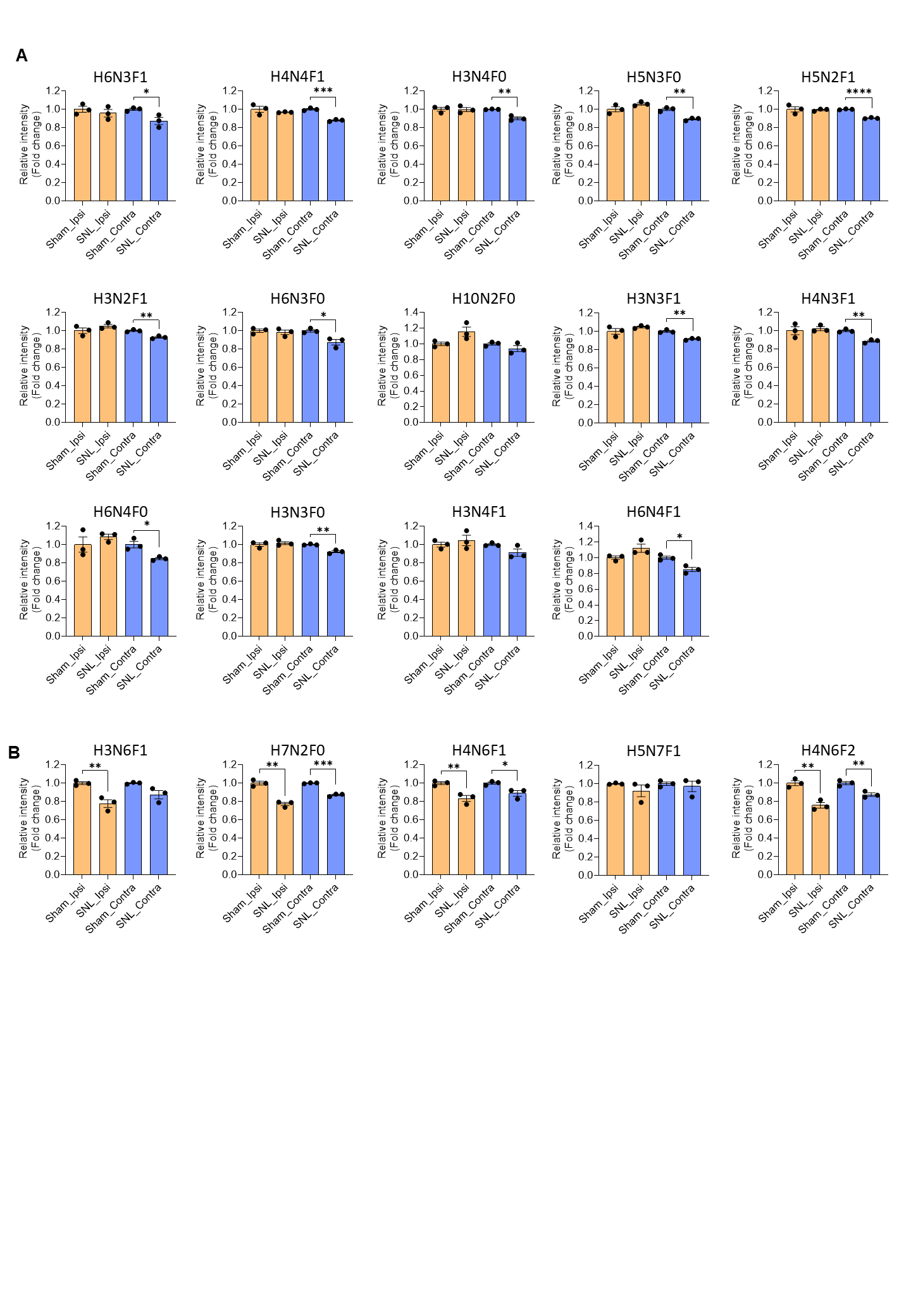
**

**Supplementary figure. 7** Differential analysis of N-glycan expression between ipsilateral and contralateral ventral horn within the SNL group. **A** Fourteen N-glycans with significantly increased relative intensity in the ipsilateral ventral horn of the SNL group. **B** Five N-glycans with significantly decreased relative intensity in the ipsilateral ventral horn of the SNL group. The values for Ipsilateral and contralateral SNL were expressed as fold change values ​​relative to the ipsilateral and contralateral sham groups, respectively (unpaired t-test, **p*<0.05, ***p*<0.01, ****p*<0.001, *****p*<0.0001, error bar: SEM, H: Hexose, N: N-acetylglucosamine, and F: fucose). Data are representative of *n*=3 independent experiments


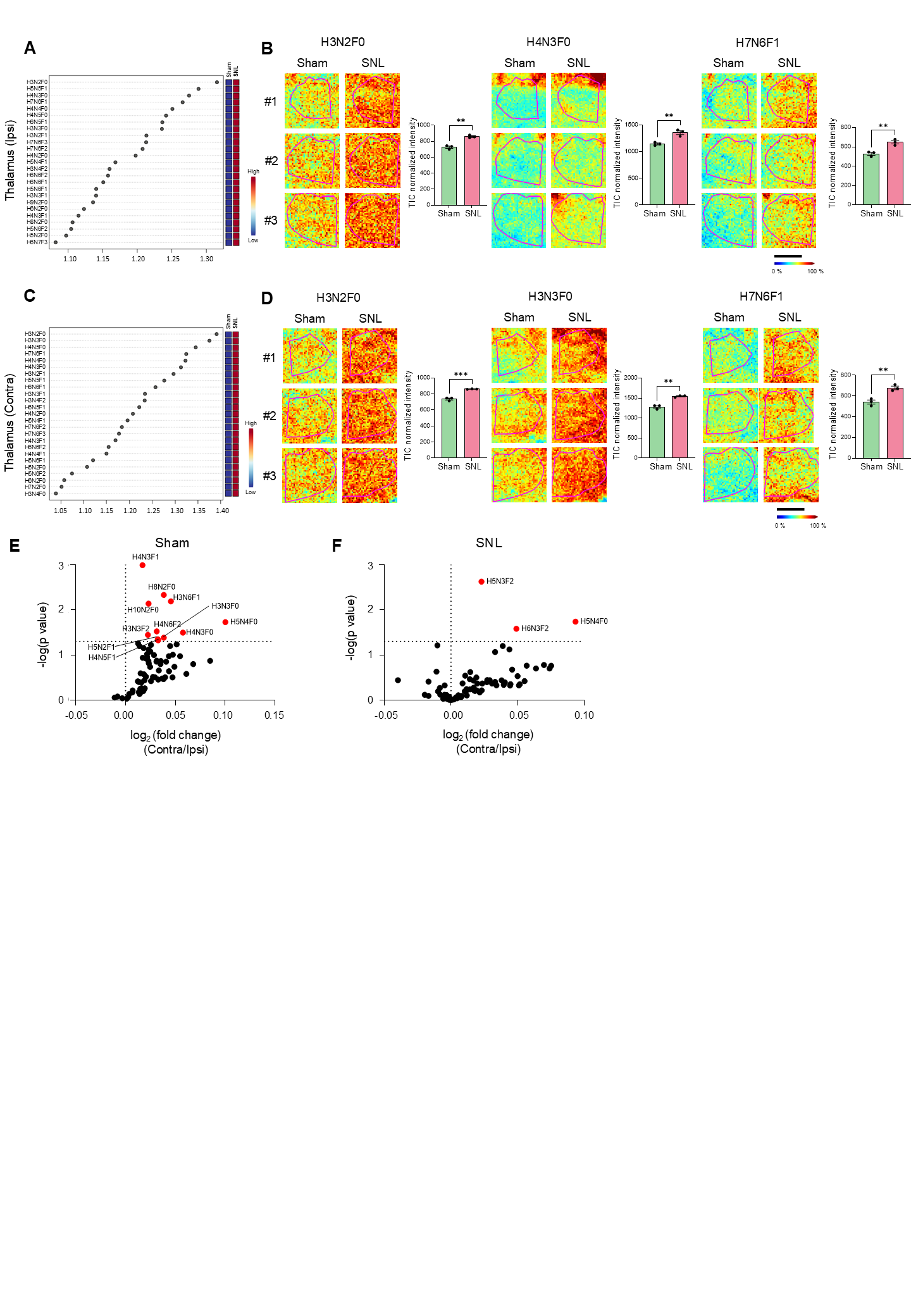


**Supplementary figure. 8** Expression changes in N-glycan in the thalamus of the brain. **A-D** Variable importance in projection (VIP) score plot MALDI mass spectrometry images with intensity plot for ipsilateral (**A**, **B**) and contralateral (**C**, **D**) thalamus. The VIP score highlights the top 25 N-glycans that contributed to the differences between the sham and SNL groups in the PLS-DA plot. The mass spectrometry images of the N-glycans with high VIP scores are presented. (unpaired t-test, ***p*<0.01, ****p*<0.001, error bar: SEM, H: Hexose, N: N-acetylglucosamine, and F: fucose). Scale bars = 2 mm. **E-F** Volcano plot of the thalamus for sham (**E**) and SNL (**F**). Data are representative of *n*=3 independent experiments


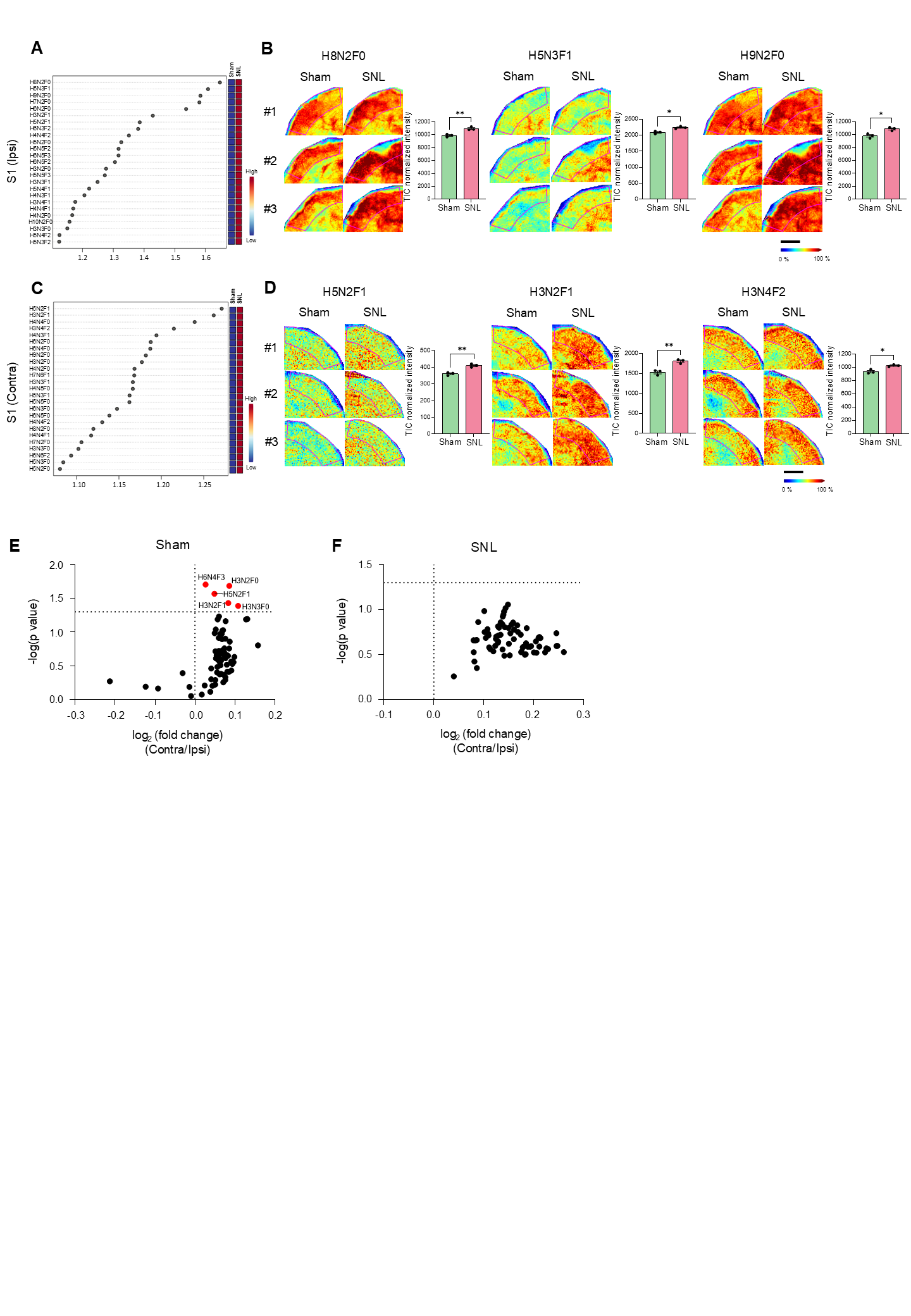


**Supplementary figure. 9** Expression changes in N-glycan in the primary sensory cortex (S1) of the brain. **A-D** Variable importance in projection (VIP) score plot MALDI mass spectrometry images with intensity plot for ipsilateral (**A**, **B**) and contralateral (**C**, **D**) S1. The VIP score highlights the top 25 N-glycans that contributed to the differences between the sham and SNL groups in the PLS-DA plot. The mass spectrometry images of the N-glycans with high VIP scores are presented (unpaired t-test, **p*<0.05, ***p*<0.01, error bar: SEM, H: Hexose, N: N-acetylglucosamine, and F: fucose). Scale bars = 2 mm. **E-F** Volcano plot of the S1 for sham (**E**) and SNL (**F**). Data are representative of *n*=3 independent experiments


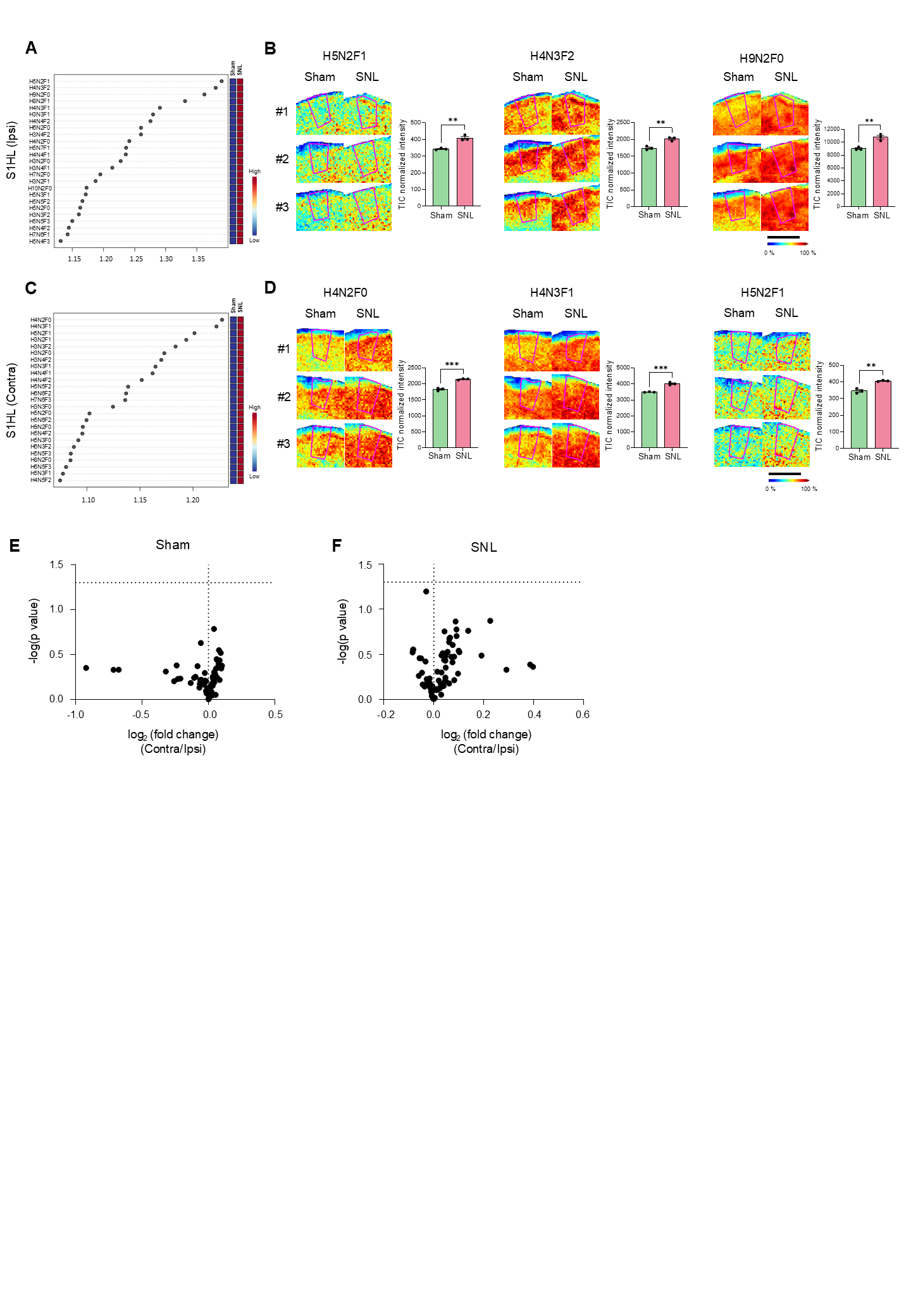


**Supplementary figure. 10** Expression changes in N-glycan in the primary sensory cortex hindlimb (S1HL) of the brain. **A-D** Variable importance in projection (VIP) score plot MALDI mass spectrometry images with intensity plot for ipsilateral (**A**, **B**) and contralateral (**C**, **D**) S1HL. The VIP score highlights the top 25 N-glycans that contributed to the differences between the sham and SNL groups in the PLS-DA plot. The mass spectrometry images of the N-glycans with high VIP scores are presented (unpaired t-test, ***p*<0.01, ****p*<0.001, error bar: SEM, H: Hexose, N: N-acetylglucosamine, and F: fucose). Scale bars = 2 mm. **E-F** Volcano plot of the S1HL for sham (**E**) and SNL (**F**). Data are representative of *n*=3 independent experiments


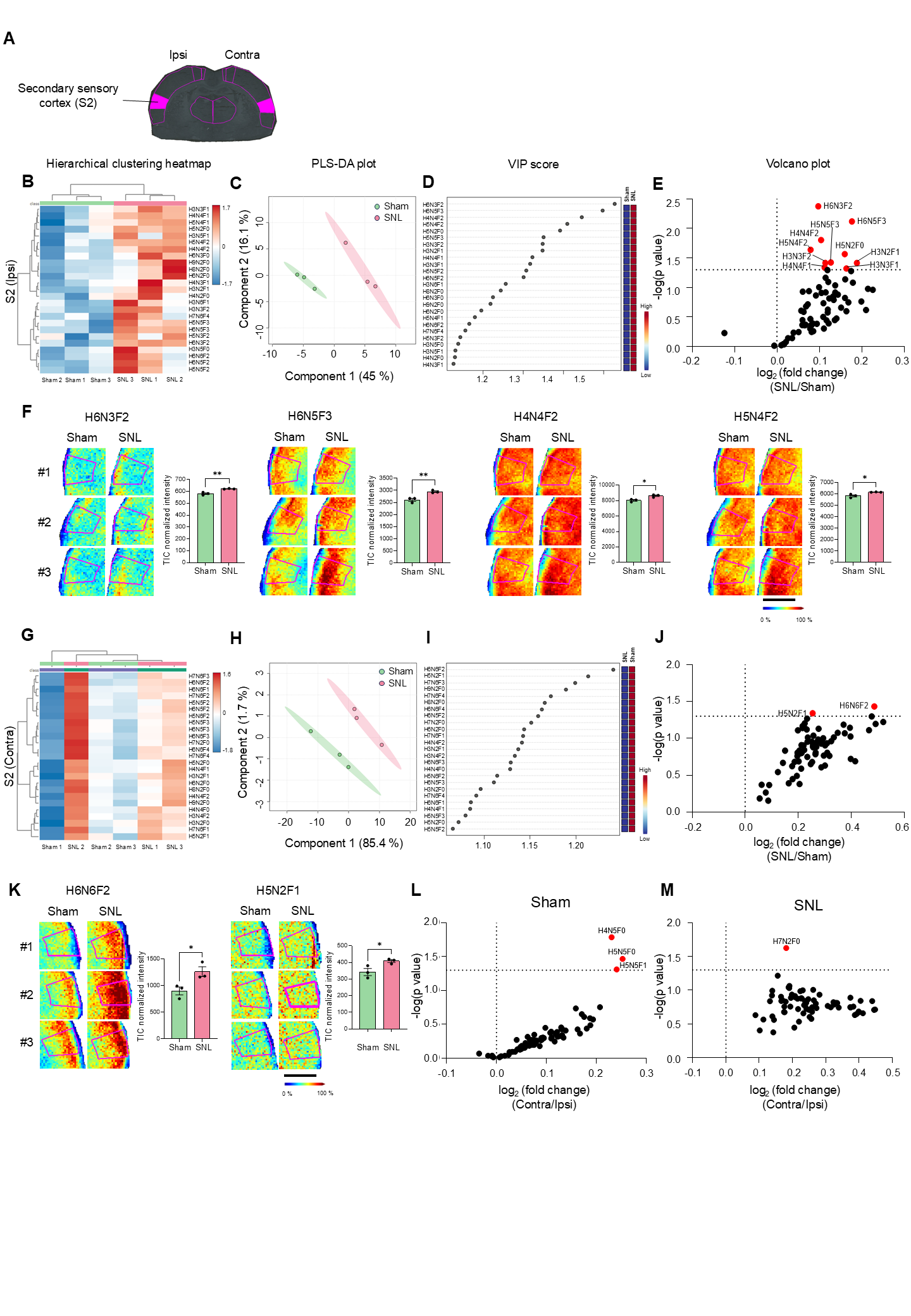


**Supplementary figure. 11** Expression changes in N-glycan in the secondary sensory cortex (S2) of the SNL brain. **A** Region of interest (ROI) of S2. **B-K** Hierarchical clustering heatmap, PLS-DA plot, VIP score plot, volcano plot, and MALDI mass spectrometry images with intensity plot for ipsilateral (**B**-**F**) and contralateral (**G**-**K**) S2. The correlation of the top 25 N-glycans detected in three samples from sham (green) and SNL (red) groups is shown in the hierarchical clustering heatmap (H: Hexose, N: N-acetylglucosamine, and F: Fucose). The PLS-DA plot of N-glycans of sham and SNL groups with 95% confidence region. The VIP score highlights the top 25 N-glycans that contributed to the differences between the sham and SNL groups in the PLS-DA plot. In the volcano plot, *p*<0.05 was considered statistically significant. Representative MALDI mass spectrometry images and intensity plots of N-glycans show significant differences between sham and SNL groups (unpaired t-test, **p*<0.05, ***p*<0.01, error bar: SEM). The MS images of the N-glycans with high VIP scores are presented. MALDI MSI was performed on three samples from each group under the same conditions. Scale bars = 2 mm. **L-M** Volcano plot of the S2 for sham (**L**) and SNL (**M**). Data are representative of *n*=3 independent experiments

**
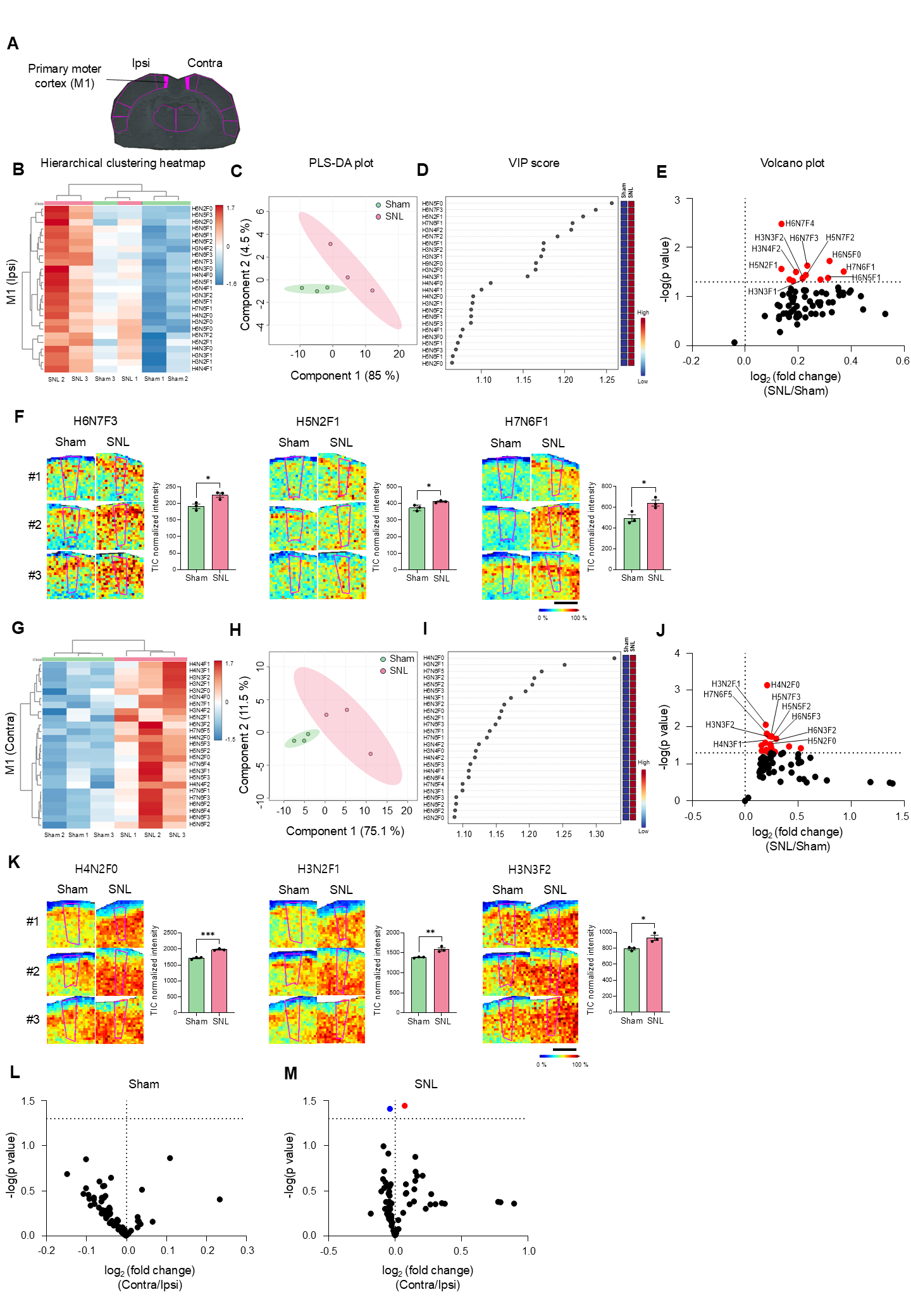
**

**Supplementary figure. 12** Expression changes in N-glycan in the primary motor cortex (M1) of the SNL brain. **A** Region of interest (ROI) of M1. **B-K** Hierarchical clustering heatmap, PLS-DA plot, VIP score plot, volcano plot, and MALDI mass spectrometry images with intensity plot for ipsilateral (**B**-**F**) and contralateral (**G**-**K**) M1. The correlation of the top 25 N-glycans detected in three samples from sham (green) and SNL (red) groups is shown in the hierarchical clustering heatmap (H: Hexose, N: N-acetylglucosamine, and F: Fucose). The PLS-DA plot of N-glycans of sham and SNL groups with 95% confidence region. The VIP score highlights the top 25 N-glycans that contributed to the differences between the sham and SNL groups in the PLS-DA plot. In the volcano plot, *p*<0.05 was considered statistically significant. Representative MALDI mass spectrometry images and intensity plots of N-glycans reveal significant differences between sham and SNL groups (unpaired t-test, **p*<0.05, ***p*<0.01, ****p*<0.001, error bar: SEM). The MS images of the N-glycans with high VIP scores are presented. MALDI MSI was performed on three samples in each group under the same conditions. Scale bars = 2 mm. **L-M** Volcano plot of the M1 for sham (**L**) and SNL (**M**). Data are representative of *n*=3 independent experiments


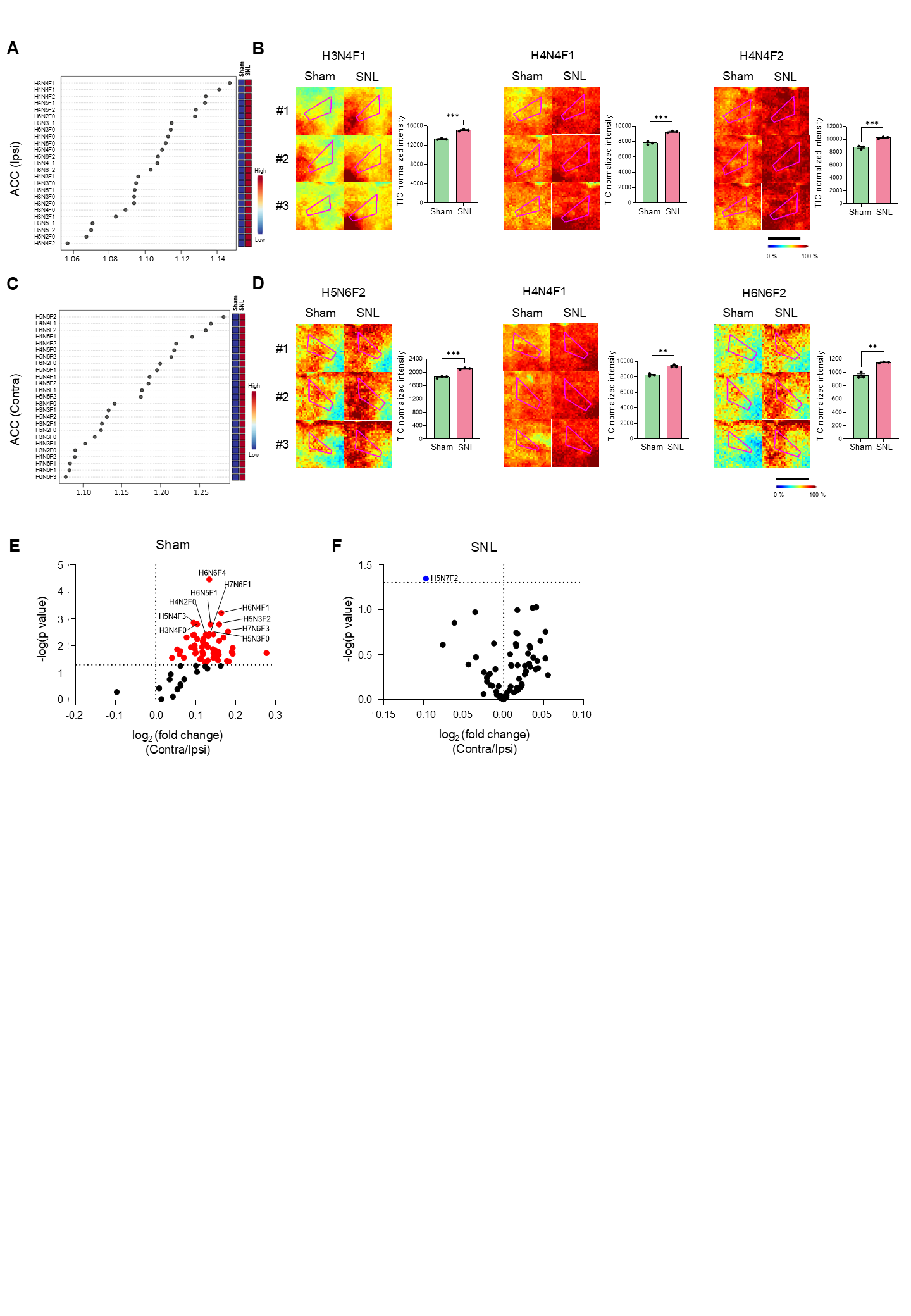


**Supplementary figure. 13** Expression changes in N-glycan in the anterior cingulate cortex (ACC) of the brain. **A-D** Variable importance in projection (VIP) score plot MALDI mass spectrometry images with intensity plot for ipsilateral (**A**, **B**) and contralateral (**C**, **D**) ACC. The VIP score highlights the top 25 N-glycans that contributed to the differences between the sham and SNL groups in the PLS-DA plot. The mass spectrometry images of the N-glycans with high VIP scores are presented (unpaired t-test, ***p*<0.01, ****p*<0.001, error bar: SEM, H: Hexose, N: N-acetylglucosamine, and F: fucose). Scale bars = 2 mm. **E-F** Volcano plot of the ACC for sham (**E**) and SNL (**F**). Data are representative of *n*=3 independent experiments

**
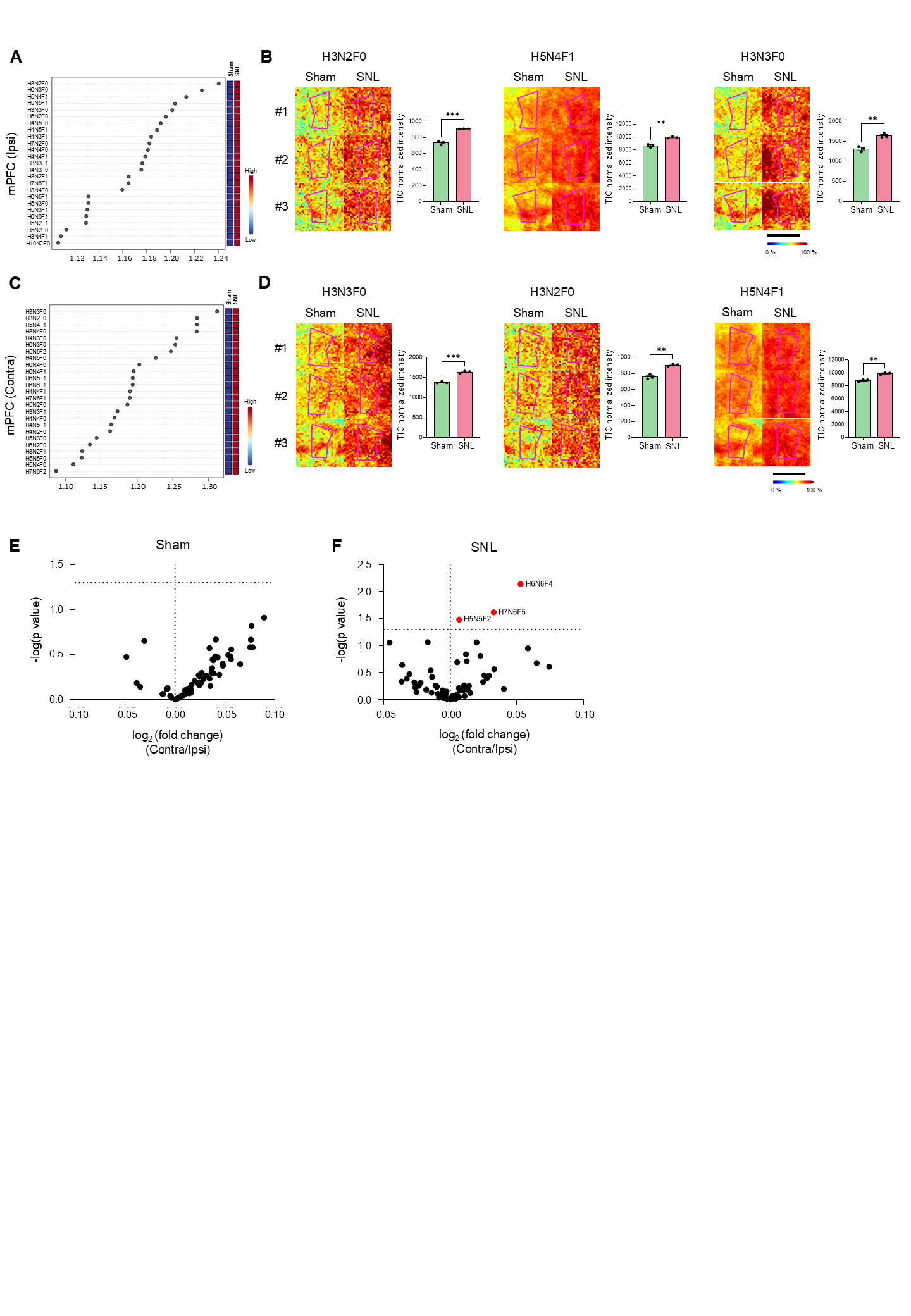
**

**Supplementary figure. 14** Expression changes in N-glycan in the medial prefrontal cortex (mPFC) of the brain. **A-D** Variable importance in projection (VIP) score plot MALDI mass spectrometry images with intensity plot for ipsilateral (**A**, **B**) and contralateral (**C**, **D**) mPFC. The VIP score highlights the top 25 N-glycans that contributed to the differences between the sham and SNL groups in the PLS-DA plot. The mass spectrometry images of the N-glycans with high VIP scores are presented (unpaired t-test, ***p*<0.01, ****p*<0.001, error bar: SEM, H: Hexose, N: N-acetylglucosamine, and F: fucose). Scale bars = 2 mm. **E-F** Volcano plot of the mPFC for sham (**E**) and SNL (**F**). Data are representative of *n*=3 independent experiments


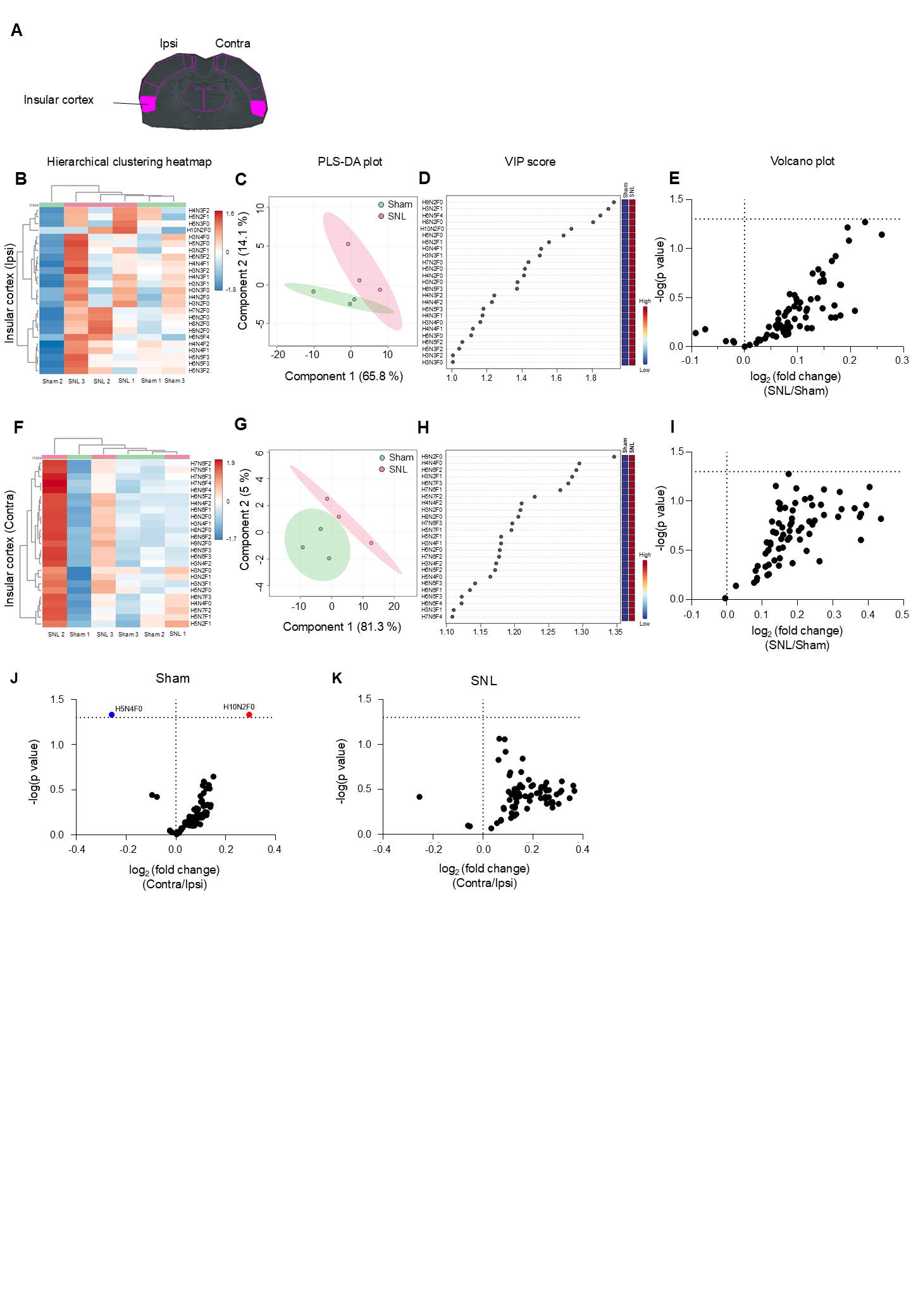


**Supplementary figure. 15** Expression changes in N-glycan in the insular cortex of the SNL brain. **A** Region of interest (ROI) of S2. **B-I** Hierarchical clustering heatmap, PLS-DA plot, VIP score plot, volcano plot, and MALDI mass spectrometry images with intensity plot for ipsilateral (**B**-**E**) and contralateral (**F**-**I**) insular cortex. The correlation of the top 25 N-glycans detected in three samples from sham (green) and SNL (red) groups is shown in the hierarchical clustering heatmap (H: Hexose, N: N-acetylglucosamine, and F: Fucose). The PLS-DA plot of N-glycans of sham and SNL groups with 95% confidence region. The VIP score highlights the top 25 N-glycans that contributed to the differences between the sham and SNL groups in the PLS-DA plot. Mass spectrometry imaging was performed on three samples in each group under the same conditions. **J-K** Volcano plot of the insular cortex for sham (**J**) and SNL (**K**). Data are representative of *n*=3 independent experiments

**
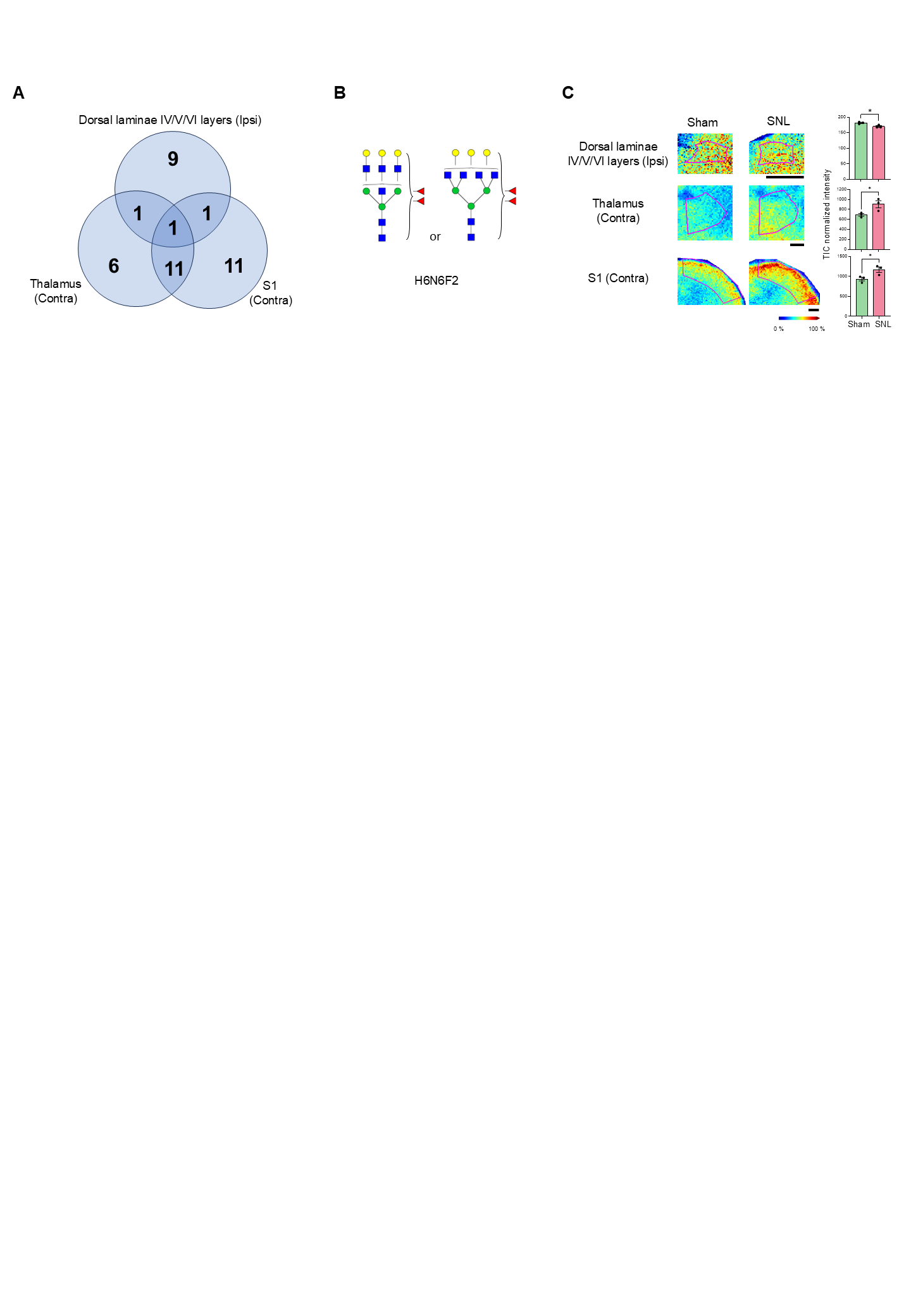
**

**Supplementary figure. 16** Significantly altered N-glycans in the spinothalamic tract. **A** Overlap of the number of significantly altered N-glycans in the three regions: ipsilateral dorsal laminae IV/V/VI layers, contralateral Thalamus, and S1, associated with the spinothalamic tract. **B** Proposed N-glycan structure of H6N6F2, represented by symbols: hexose (H), mannose (green circle), galactose (yellow circle), N-acetylglucosamine (GlcNAc, N, blue square), and fucose (F, red triangle). **C** Mass spectrometry images and intensity plots of H6N6F2 in the three regions (unpaired t-test, **p*<0.05, error bar: SEM, H: Hexose, N: N-acetylglucosamine, and F: fucose). Scale bars = 1 mm. Data are representative of *n*=3 independent experiments**
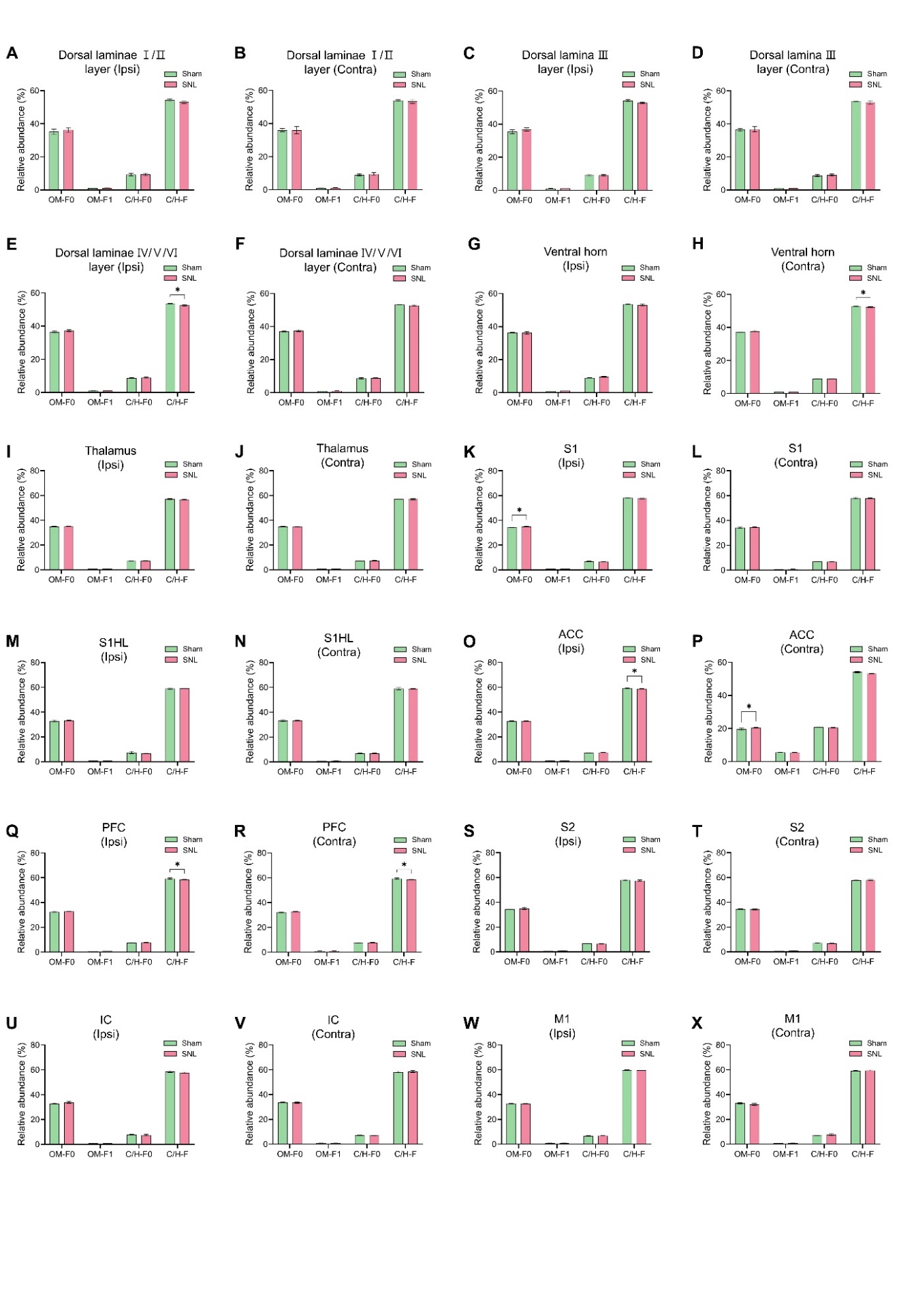
**

**Supplementary figure 17.** Comparison of relative abundance between N-glycan types in the spinal cord and brain region. The N-glycans detected in the ipsilateral and contralateral tissues of the dorsal laminae Ⅰ/Ⅱ (**A**-**B**), Ⅲ (**C**-**D**), Ⅳ/Ⅴ/Ⅵ layers (**E-F**), ventral horn (**G**-**H**), thalamus (**I**-**J**), S1 (**K**-**L**), S1HL (**M**-**N**), ACC (**O**-**P**), PFC (**Q-R**), S2 (**S**-**T**), IC (**U**-**V**), and M1 (**W**-**X**) were classified according to their biosynthetic classes and degree of fucosylation. The percentage of relative abundance was calculated by dividing the total N-glycan intensity by the N-glycan intensities in each class (OM: Oligomannose, C/H: complex/hybrid, F0: zero fucosylated, F1: mono fucosylated, F: all fucosylated glycans, two-way ANOVA with tukey’s post hoc, **p*<0.05). Data are representative of *n*=3 independent experiments

**Supplementary Tables**





**Supplementary Table. 1** A list of primers used for real-time PCR


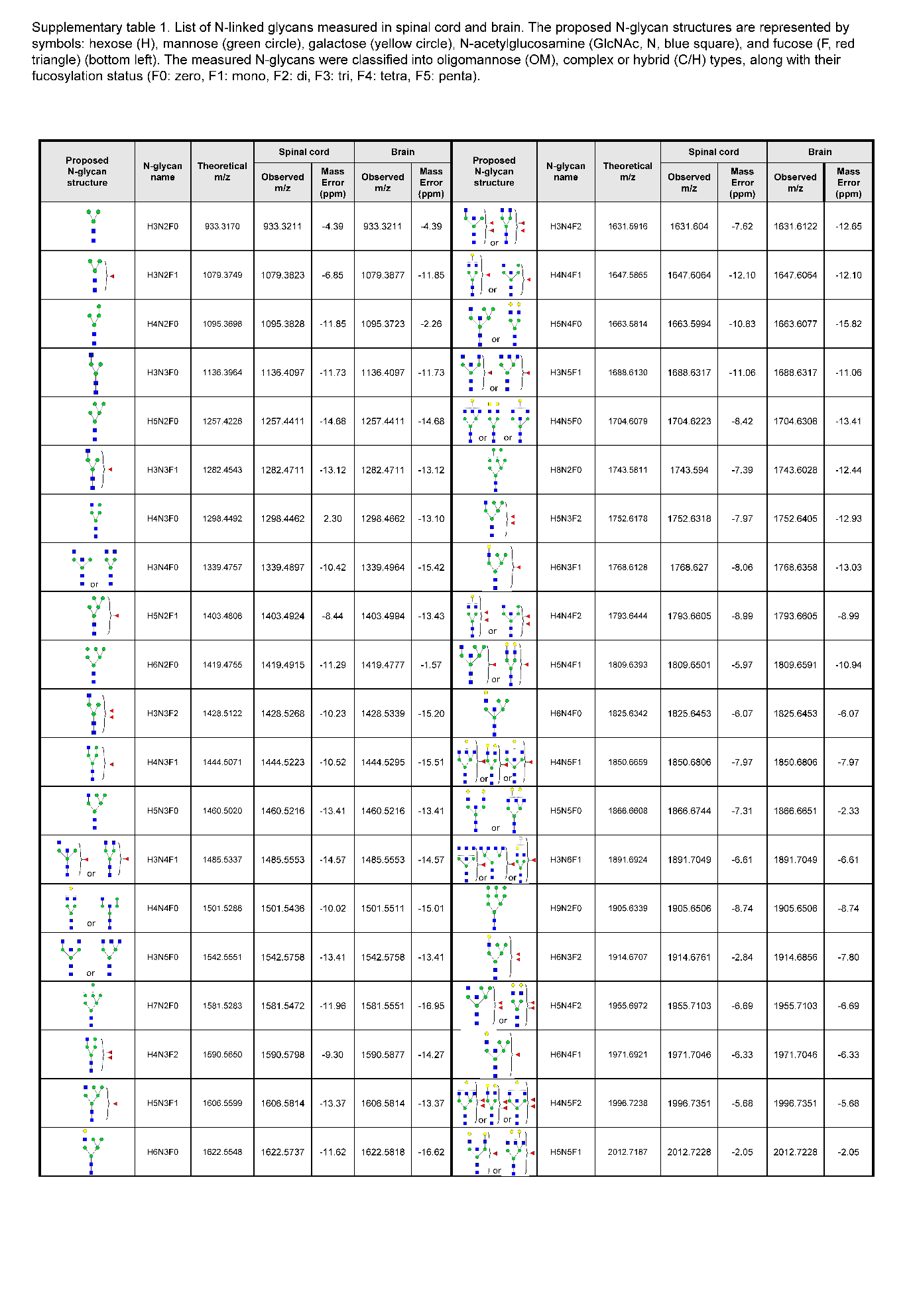


**
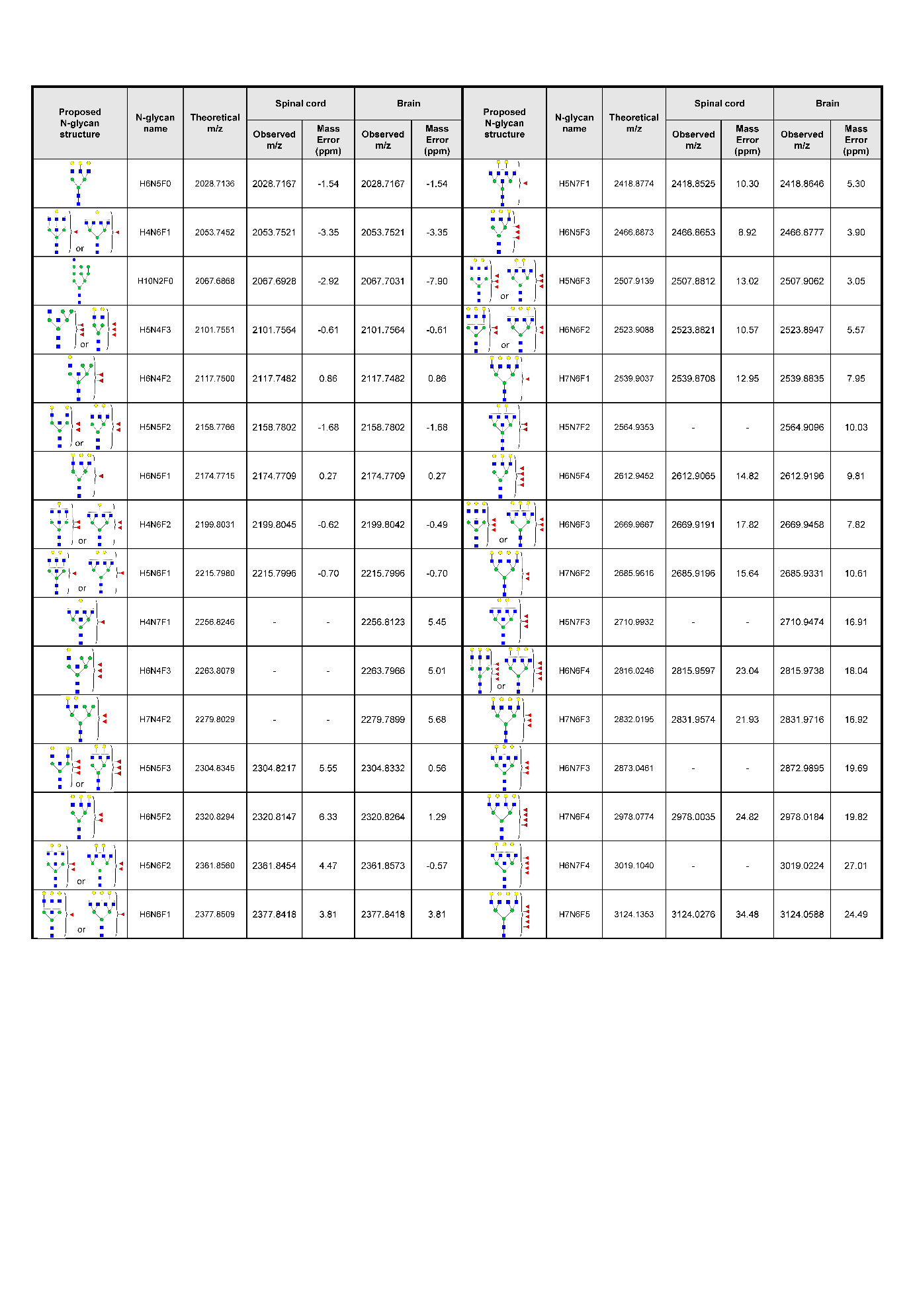
Supplementary table. 2** A list of N-linked glycans measured in spinal cord and brain. The proposed N-glycan structures are represented by symbols: hexose (H), mannose (green circle), galactose (yellow circle), N-acetylglucosamine (GlcNAc, N, blue square), and fucose (F, red triangle) (bottom left). The measured N-glycans were classified into oligomannose (OM), complex or hybrid (C/H) types, along with their fucosylation status (F0: zero, F1: mono, F2: di, F3: tri, F4: tetra, F5: penta)

**
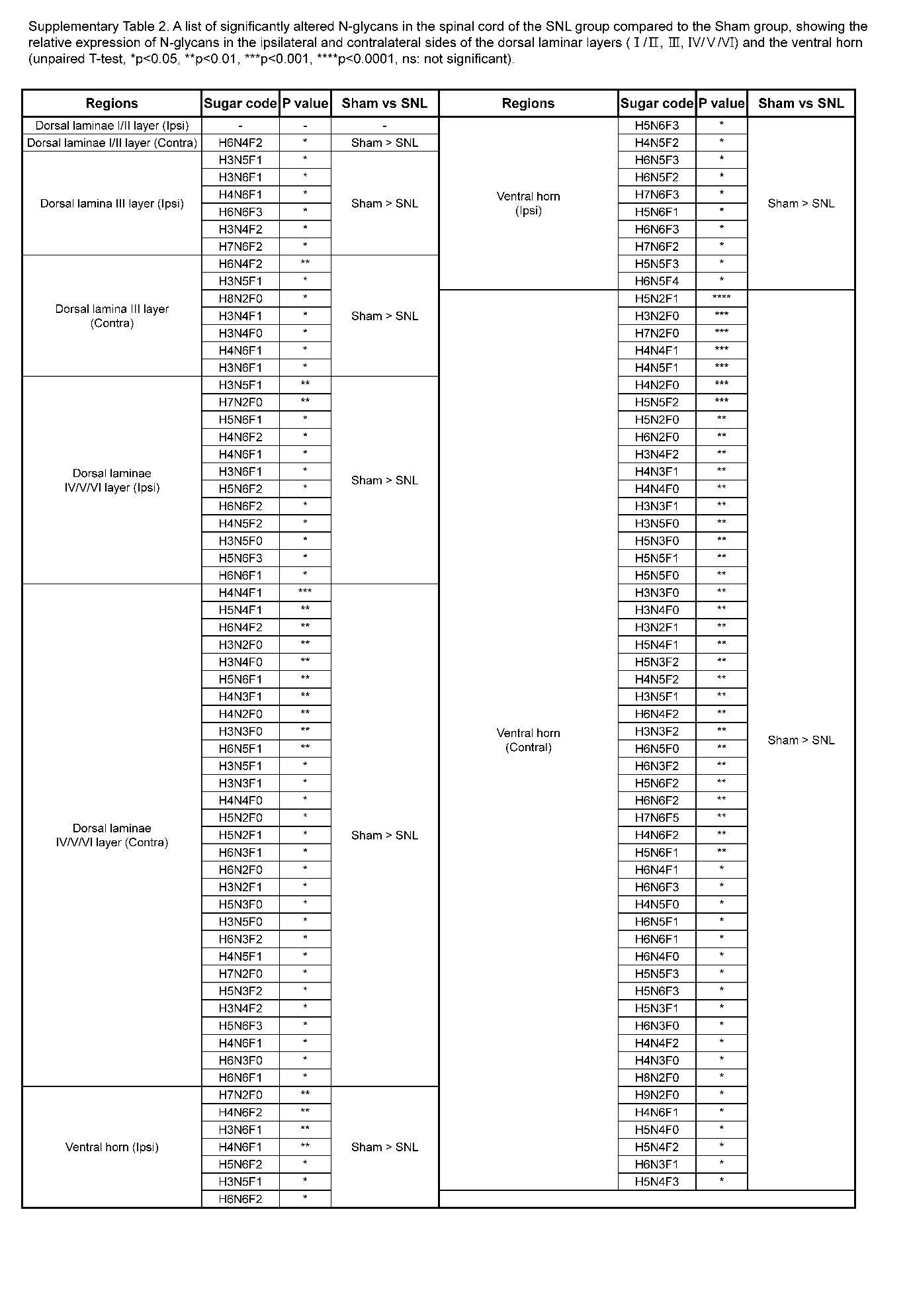
**

**Supplementary Table. 3** A list of significantly altered N-glycans in the spinal cord of the SNL group compared to the sham group. The relative expression of N-glycans in the ipsilateral and contralateral sides of the dorsal laminar layers (I/II, III, IV/V/VI) and the ventral horn (unpaired t-test, **p*<0.05, ***p*<0.01, ****p*<0.001, *****p*<0.0001, ns: not significant)


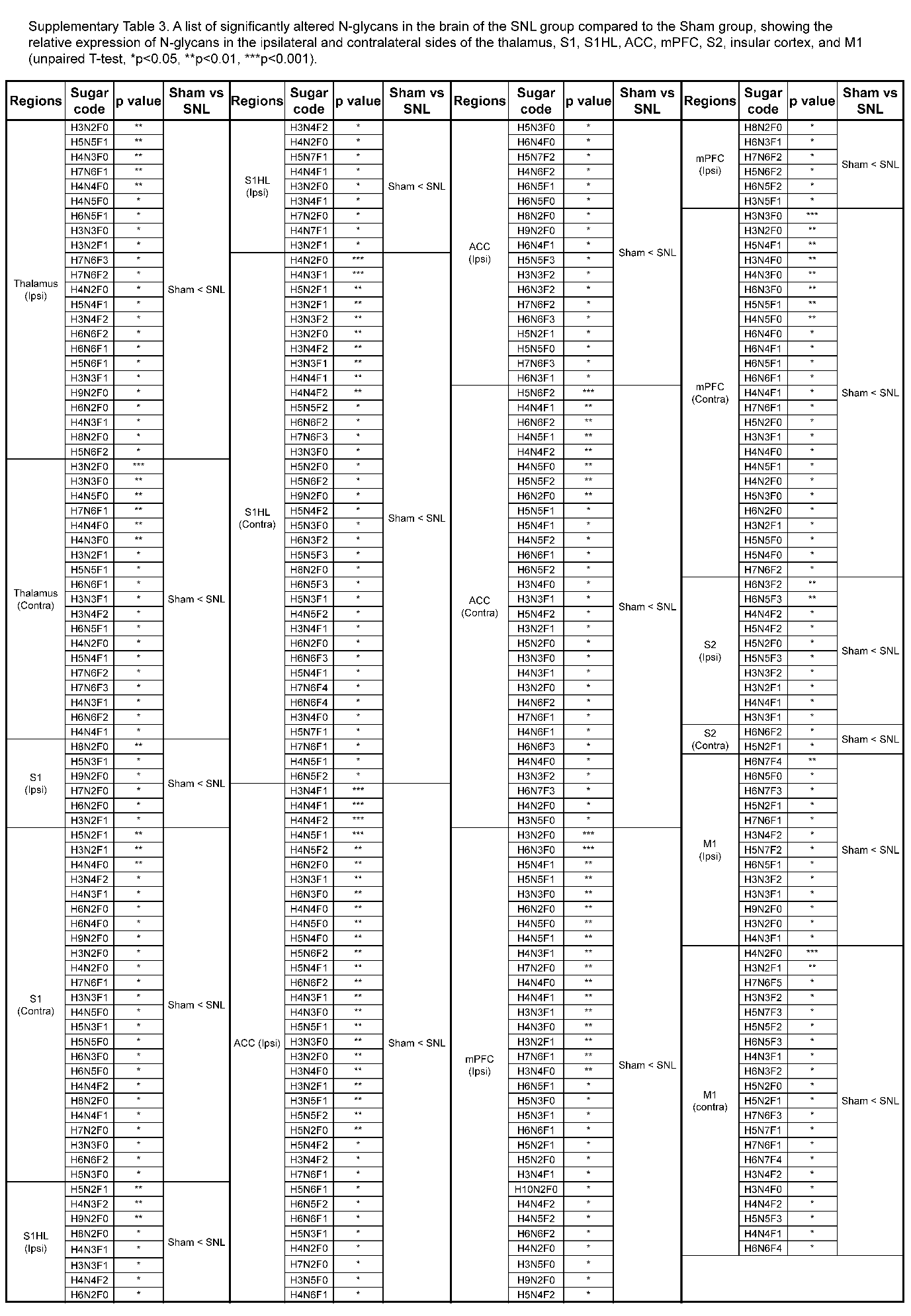


**Supplementary Table. 4** A list of significantly altered N-glycans in the brain of the SNL group compared to the sham group. The relative expression of N-glycans in the ipsilateral and contralateral sides of the thalamus, S1, S1HL, ACC, mPFC, S2, insular cortex, and M1 (unpaired t-test, **p*<0.05, ***p*<0.01, ****p*<0.001).


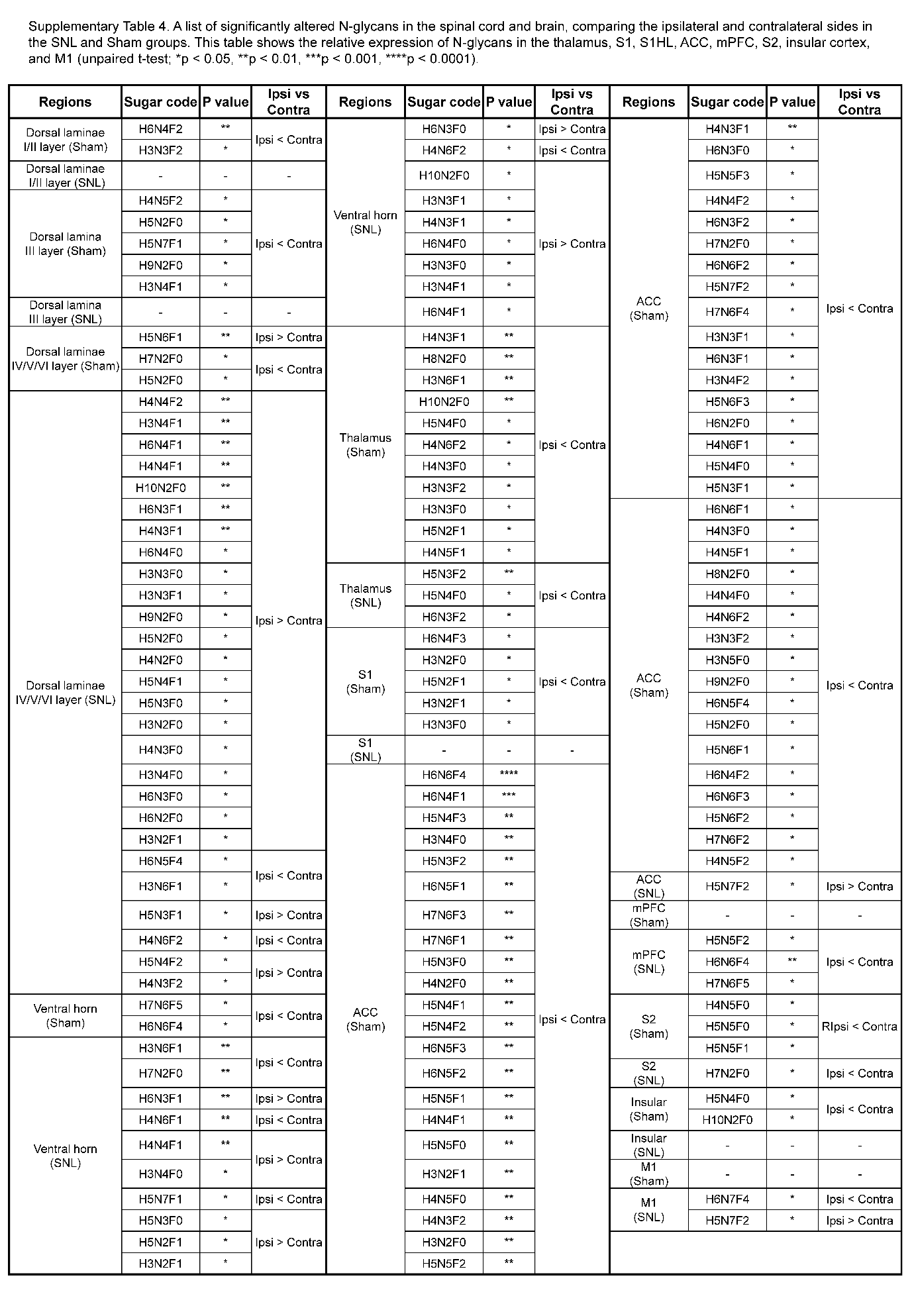


**Supplementary Table. 5** A list of significantly altered N-glycans in the spinal cord and brain, comparing the ipsilateral and contralateral sides in the SNL and sham groups. This table shows the relative expression of N-glycans in the thalamus, S1, S1HL, ACC, mPFC, S2, insular cortex, and M1 (unpaired t-test; **p* < 0.05, ***p* < 0.01, ****p* < 0.001, *****p* < 0.0001)
